# Supplementary material for: MicroRNA‐483 amelioration of experimental pulmonary hypertension
Source: EMBO Mol Med. 2020 Apr 23;12(5):e11303. doi: 10.15252/emmm.201911303 (PMC7207157; doi:10.15252/emmm.201911303)
Supplement: Supplementary file 1 — Appendix [file EMMM-12-e11303-s001.pdf]

Appendix

Table of contents

- **Appendix figure S1**
- **Appendix figure S2**
- **Appendix figure S3**
- **Appendix figure S4**
- **Appendix figure S5**
- **Appendix figure S6**
- **Appendix figure S7**
- **Appendix figure S8**
- **Appendix figure S9**
- **Appendix figure S10**
- **Appendix figure S11**
- **Appendix figure S12**
- **Appendix figure S13**
- **Appendix figure S14**
- **Appendix table S1**
- **Appendix table S2**
- **Appendix table S3**

Appendix Figure S1

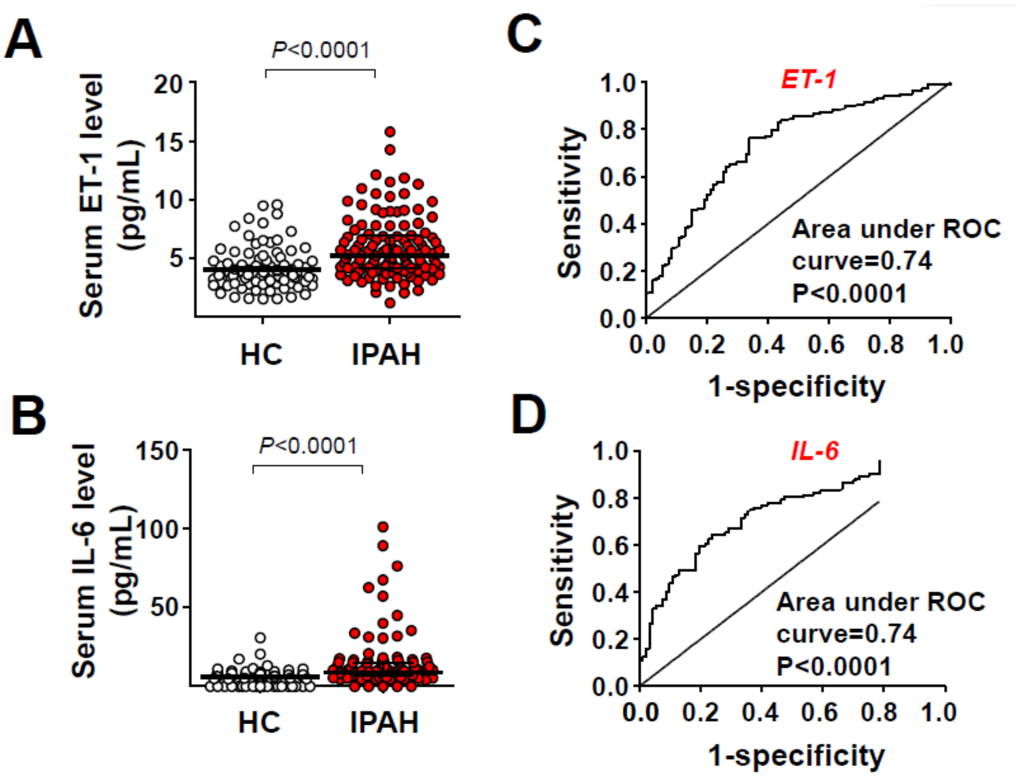

Figure S1. Elevated ET-1 and IL-6 levels in IPAH serum.

(A, B) Serum levels of ET-1 (IPAH, n=118; HC, n=93) (**A**) and IL-6 (IPAH, n=112; HC, n=93) (**B**) measured by ELISA.

(C, D) Receiver operating characteristic (ROC) curve of ET-1 (**C**) and IL-6 (**D**).

Data information: Values are expressed as median ± interquartile range. Statistical test: Mann-Whitney *U* test.

Appendix Figure S2

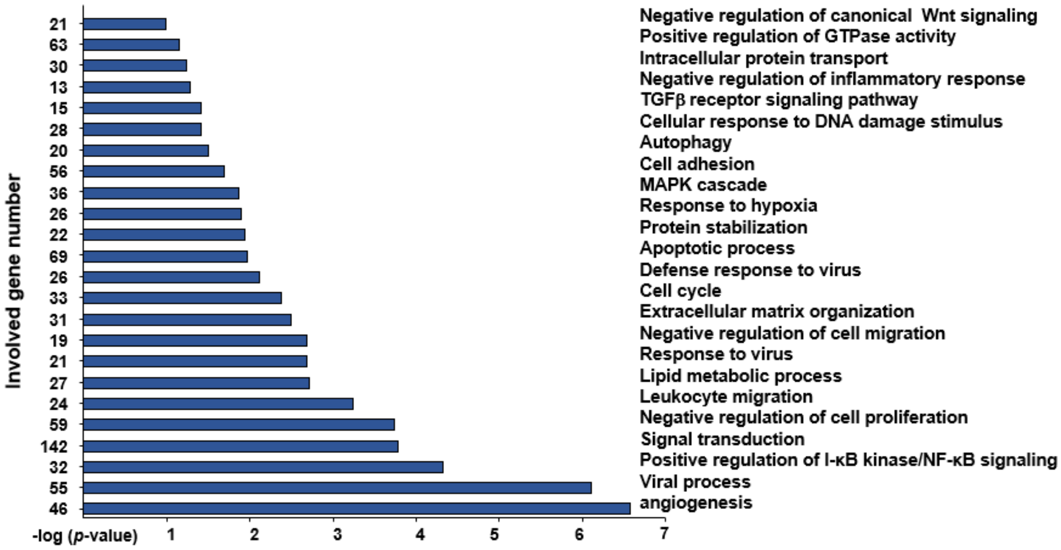

Figure S2. miR-483 overexpression regulates multiple signaling pathways in PAECs.

PAECs were transfected with scramble or miR-483-3p/5p mimic for 36 hr. Cells were harvested and the isolated RNA was analyzed by RNA-seq. The GO enrichment was delineated by DAVID for the top 300 up-regulated or down-regulated genes, with the cutoff of $p<0.05$. Data are results from two biological repeats.

Appendix Figure S3

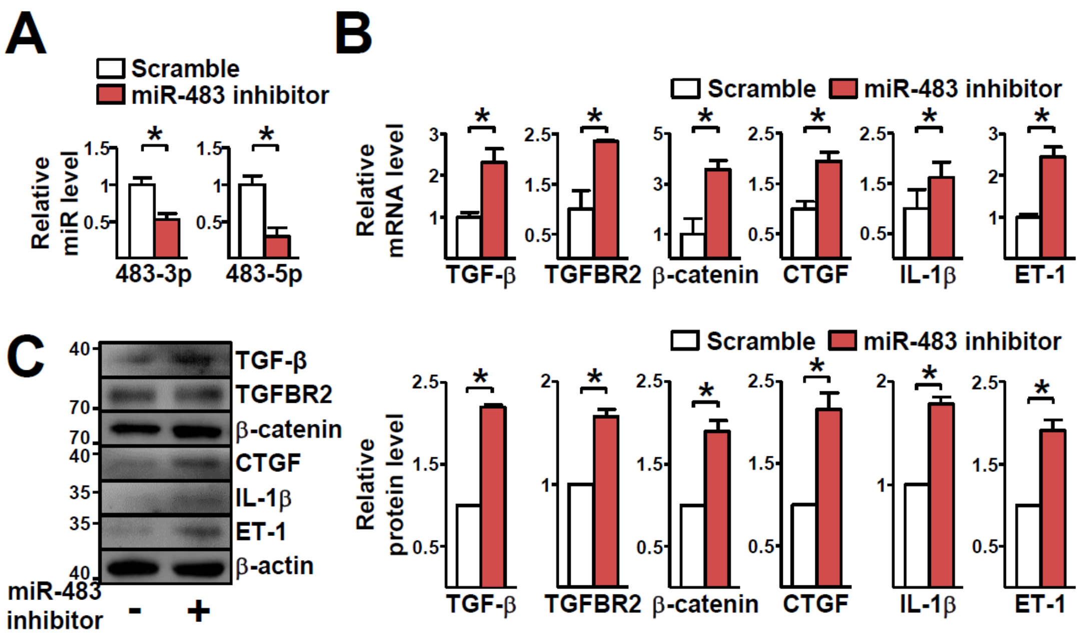

Figure S3. miR-483 inhibition increased expression of its target genes in PAECs.

PAECs were transfected with scramble or anti-miR-483 for 24 hr. Levels of miR-483-3p/-5p, TGF-β, TGFBR2, β-catenin, CTGF, Il-1β, and ET-1 mRNA and protein were measured by qPCR and Western blot, respectively.

Data information: Values are expressed as mean ± SEM from 3 independent experiments. Statistical test: *t*-test (**P* < 0.05 between the indicated groups, exact *P* values were shown in Appendix Table S3).

Appendix Figure S4

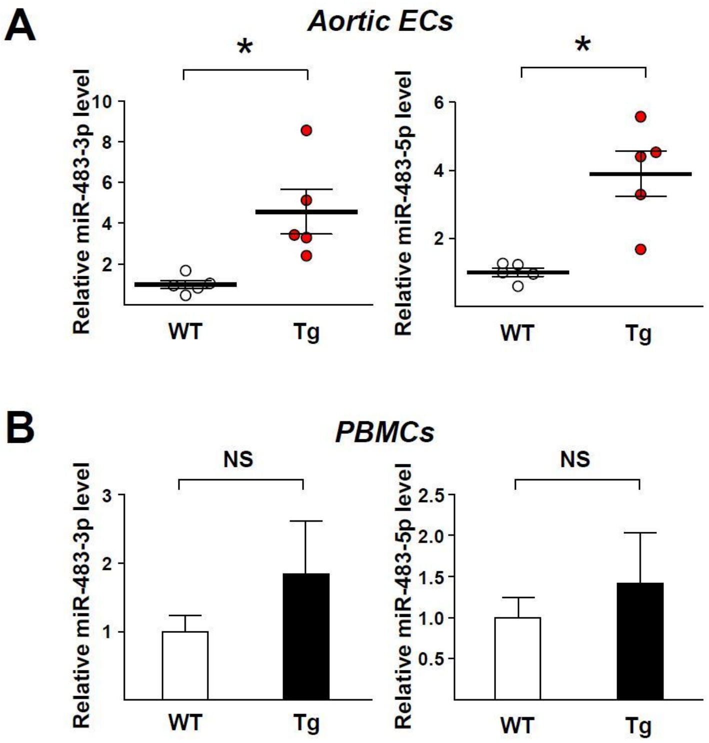

Figure S4. In EC-miR-483-Tg rats, miR-483 was increased in aortic ECs but not in PBMCs.

(A, B) Aortic ECs (**A**) and PBMCs (**B**) were isolated from EC-miR-483-Tg rats and their wile-type (WT) littermates, miR-483-3p/-5p levels were measured by RT-qPCR.

Data information: Values are expressed as mean ± SEM. Five rats (**A**) and three rats (**B**) were used for the indicated experiments. Statistical test: *t* test (*$P < 0.05$ between the indicated groups, exact *P* values were shown in Appendix Table S3).

Appendix Figure S5

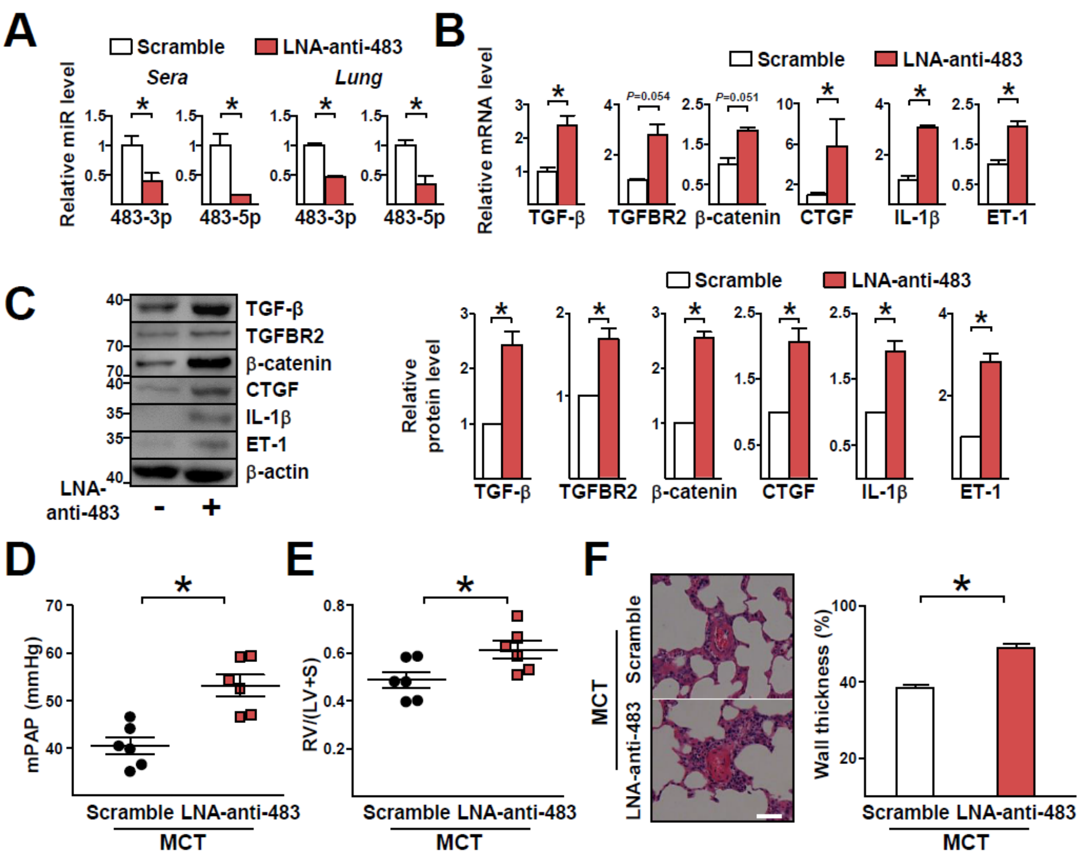

Figure S5. MiR-483 inhibition exacerbated MCT-induced PH.

MCT-treated rats were intratracheally delivered LNA-miR-483-3p and -5p or scramble at Day 21 and Day 28 post the MCT treatment (M+Scramble, M+LNA-483).

(A-C) qPCR analysis of miR-483-3p/-5p in serum and lung tissue from PH (MCT-treated) rats (**A**). mRNA (**B**) and protein levels (**C**) of TGF-β, TGFBR2, β-catenin, CTGF, IL-1β, and ET-1 measured by qPCR and Western blotting, respectively (n=2×3, proteins were pooled from two animals for each experiment and there were 3 independent experiments).

(D-F) show the measured mPAP; RV hypertrophy [RV/(LV+S)]; and pulmonary arterial wall thickness for the indicated groups (n=6 in each group).

Data information: Values are expressed as mean ± SEM. Statistical test: *t*-test (**P* < 0.05 between the indicated groups, exact *P* values were shown in Appendix Table S3). Scale bar: 20 µm.

Appendix Figure S6

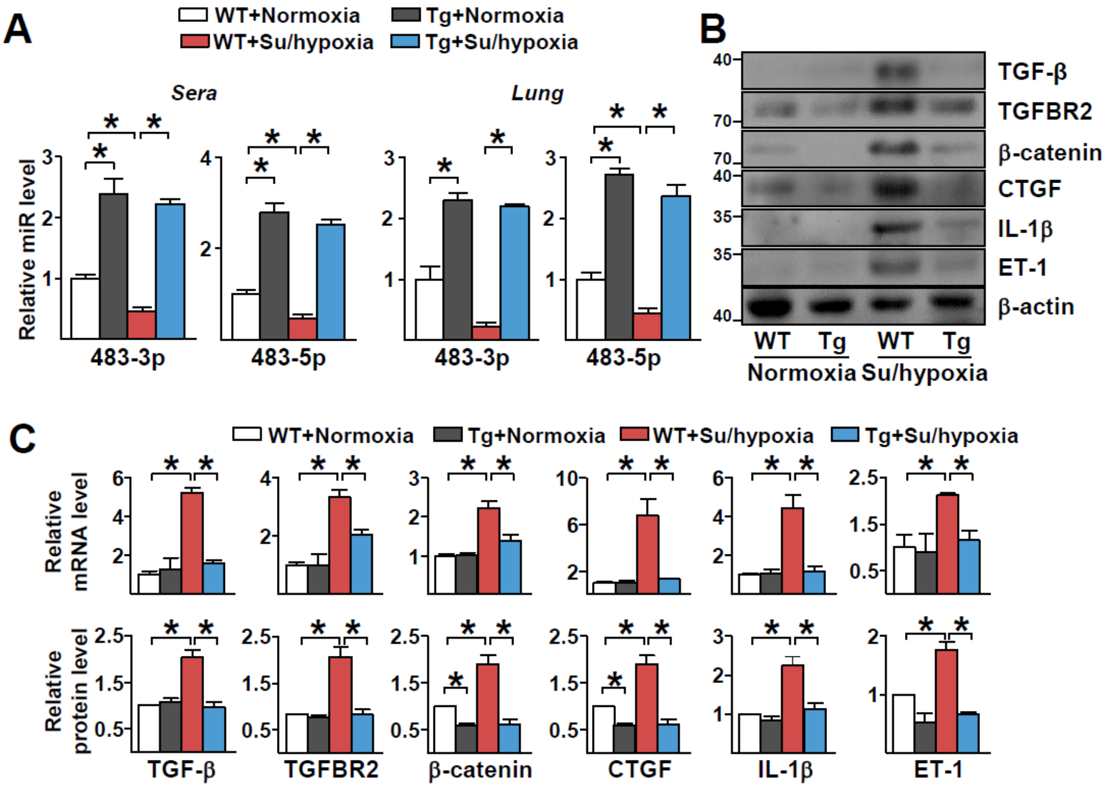

Figure S6. Decreased expression of PAH-related genes in SU5416/hypoxia-treated EC-miR-483-Tg rats.

EC-miR-483-Tg rats and WT littermates were administered SU5416, exposed to hypoxia for 3 weeks, and then reoxygenation for 2 weeks or injected with DMSO and exposed to normoxia for 5 weeks.

(A) Levels of miR-483-3p/-5p in serum and lung tissues were measured by qPCR.

(B, C) Expression levels of TGF-β, TGFBR2, β-catenin, CTGF, IL-1β, and ET-1 mRNA and protein in lung tissues were measured by qPCR and Western blot, respectively.

Data information: Values are expressed as mean ± SEM. n=2×3 samples were pooled from two animals for each experiment and there were 3 independent experiments. Statistical test: *t*-test or ANOVA (**P* < 0.05 or **P* < 0.05/Bonferroni between the indicated groups, exact *P* values were shown in Appendix Table S3).

Appendix Figure S7

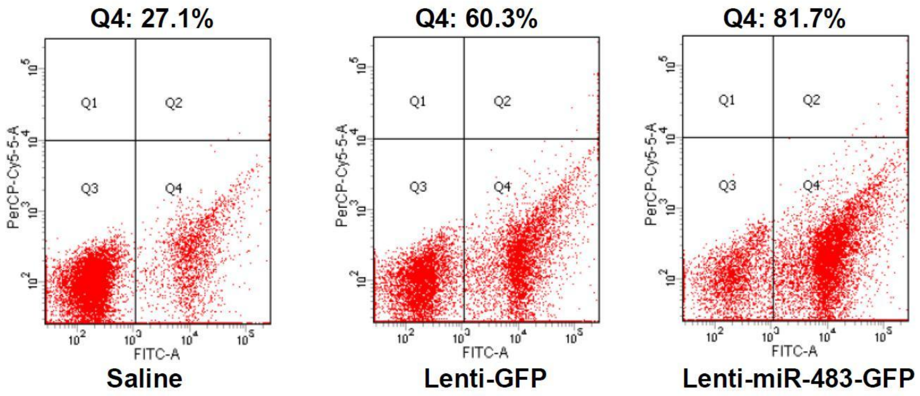

Figure S7. Efficiency of lentivirus inhalation.

Lung tissues from male SD rats receiving lentivirus-GFP or lentivirus-miR-483-GFP were digested and analyzed by flow cytometry. Cells with GFP expression were detected and analyzed by BD FACSAria III system.

Appendix Figure S8

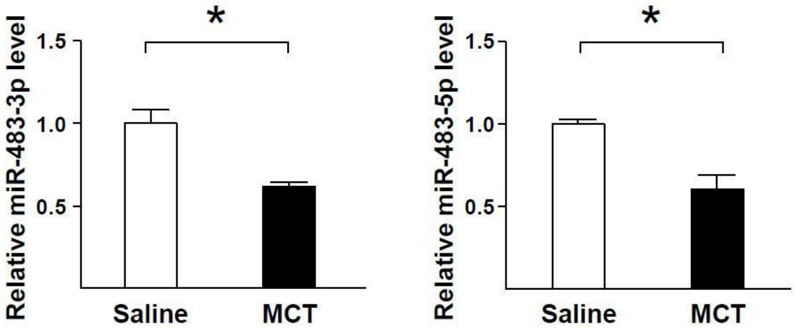

Figure S8. Decreased miR-483 in lung ECs of MCT-PH rats.

Lung endothelial cells (LECs) were isolated from rats receiving MCT or saline (n=3 for each group). MiR-483-3p/-5p levels were detected by RT-qPCR.

Data information: Values are expressed as mean ± SEM. Statistical test: *t*-test (**P* < 0.05 or between the indicated groups, exact *P* values were shown in Appendix Table S3).

Appendix Figure S9

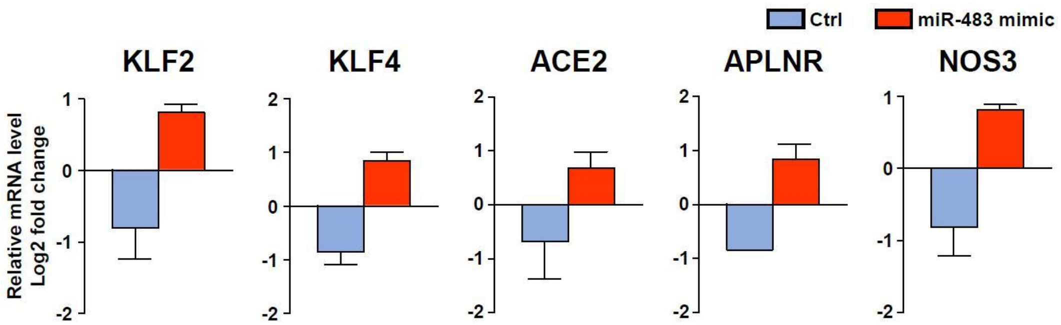

Figure S9. Overexpression of miR-483 increases expression of genes involved in EC homeostasis.

Data are results from RNA-seq experiments described in Fig.2B. Bar graphs were generated for comparison of log2-fold changes in the indicated genes.

Appendix Figure S10

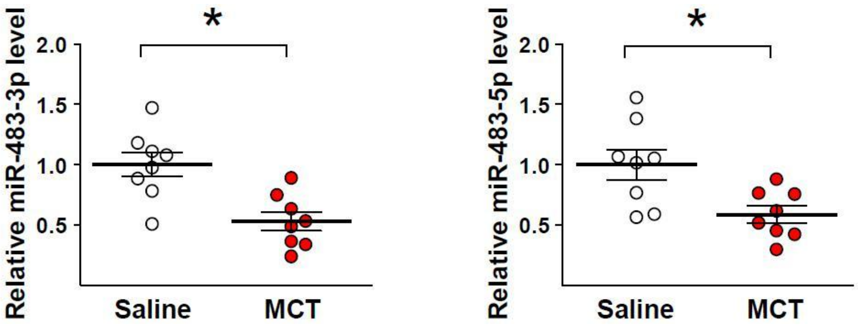

Figure S10. Reduced miR-483-3p/-5p levels in CD144-enriched EVs in serum of MCT-PH rats and control rats.

CD144-enrinched EVs were isolated from serum of rats injected with saline or MCT; miR-483-3p/-5p levels were measured by qPCR (n=8 for each group).

Data information: Values are expressed as mean ± SEM. Statistical test: *t*-test (**P* < 0.05 or between the indicated groups, exact *P* values were shown in Appendix Table S3).

Appendix Figure S11

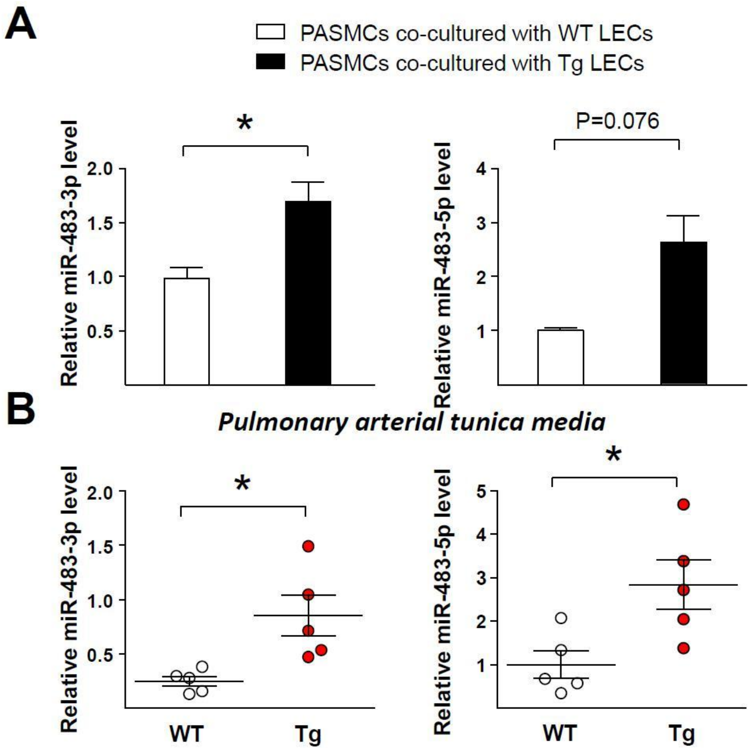

Figure S11. EC-derived miR-483 is involved in EC-VSMC communication.

(A) PASMCs were co-cultured with lung ECs isolated from EC-miR-483-Tg (Tg) rats or their wide-type littermates (WT) by a transwell system with 0.4-µm aperture. Briefly, PASMCs and lung ECs were cultured in the upper or lower chamber respectively for 24 hr.

(B) Pulmonary arterial tunica media was isolated from of EC-miR-483-Tg rats and wide-type littermates; the level of miR-483 was detected by RT-qPCR (n=5 for each group)

Data information: Values are expressed as mean ± SEM. Data are representative of three independent experiments (**A**). Statistical test: *t*-test (**P* < 0.05 or between the indicated groups, exact *P* values were shown in Appendix Table S3).

Appendix Figure S12

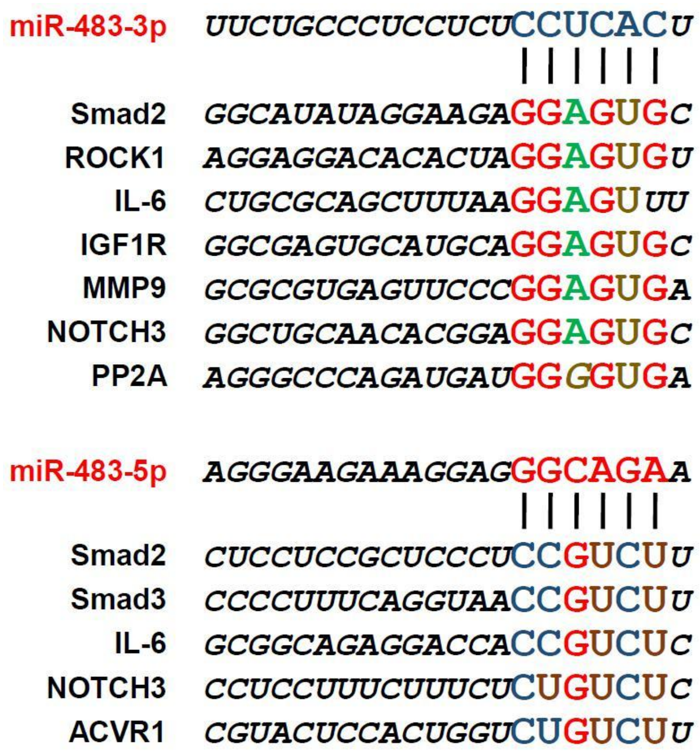

Figure S12. Other mRNAs predicted to be targeted by miR-483-3p/-5p.

The putative target sites of miR-483-3p/-5p on 3'UTR of SMAD2, SMAD3, ROCK1, IL-6, IGF1R, NOTCH3, MMP9, PP2A, and ACVR1 mRNA were predicted by RNAhybrid (Kruger and Rehmsmeier 2006).

Appendix Figure S13

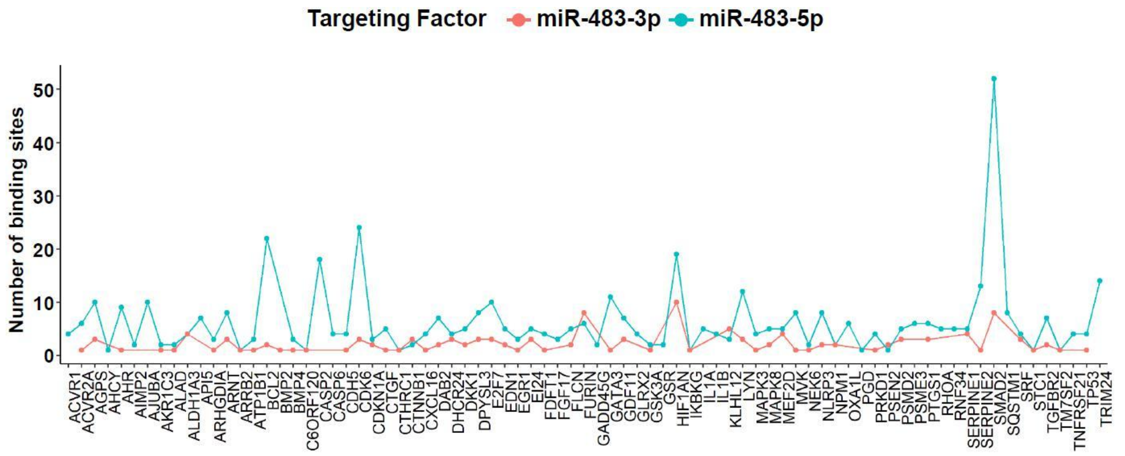

Figure S13. In silico prediction of genes targeted by miR-483-3p/-5p.

The number of miR-483-3p and miR-483-5p binding sites in the mRNA of genes deferentially regulated by miR-483 overexpression that are implicated in PAH.

Appendix Figure S14

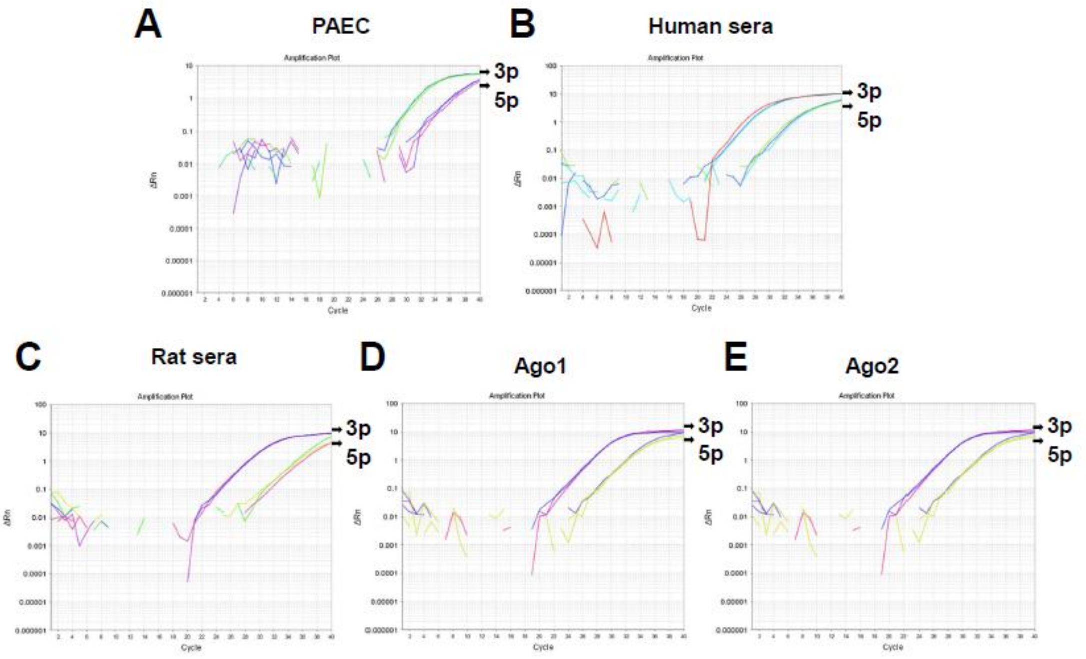

Figure S14. The qPCR amplification plots of miR-483-3p and -5p.

A-E The qPCR amplification plots of miR-483-3p and -5p in PAEC (**A**), human sera (**B**), rat sera (**C**), Ago1-miRISC (**D**) and Ago2-miRISC (**E**). Three PCR samples are presented for each group.

Appendix Table S1

miR-483-3p

<table>
<tr><th>Name</th><th>Species</th><th>MFE</th><th>Site</th></tr>
<tr><td rowspan="3">TGF-β</td><td>Human</td><td>-19.8</td><td>5′ cAGGCc--AGGc--GGGGUGg 3′
|||| ||| || ||
3′ uUCUGcccUCCucuCCUCACu 5′</td></tr>
<tr><td>Mouse</td><td>-20.1</td><td>5′ cUGGGGGGcccauuaaaGGUGAc 3′
||||||| |||||
3′ uGCCCUCCccucc----UCACU 5′</td></tr>
<tr><td>Rat</td><td>-18.8</td><td>5′ ggcccauuaaaGGugacaGAGGAa 3′
|| |||||
3′ guucugcccucCC-----CUCCUc 5′</td></tr>
<tr><td rowspan="3">TGFBR2</td><td>Human</td><td>-26.4</td><td>5′ ugcauuGGAG-AGAGGAGUGc 3′
|||| |||||||||
3′ uucugcCCUCcUCUCCUCACu 5′</td></tr>
<tr><td>Mouse</td><td>-32.5</td><td>5′ gcaGCGGcGGGGGAuGAGUGAc 3′
|||| |||||| ||||||
3′ uucUGCCcUCCCCUcCUCACU 5′</td></tr>
<tr><td>Rat</td><td>-20.0</td><td>5′ AAGACcaGAGGucaGcAGGAuUGc 3′
||||| |||| | |||| ||
3′ UUCUGccCUCC-c-C-UCCUcAC 5′</td></tr>
<tr><td rowspan="3">IL-1β</td><td>Human</td><td>-17.0</td><td>5′ aGGGGGcaagaAGuAGcAGUGu 3′
||||| || || ||||
3′ gCCCUCc----UC-UCcUCACu 5′</td></tr>
<tr><td>Mouse</td><td>-17.4</td><td>5′ AAGACaGGucGcucAGGGu 3′
||||| || | ||||
3′ UUCUGcCCucCcc-UCCUcacu 5′</td></tr>
<tr><td>Rat</td><td>-22.5</td><td>5′ auucaGGGGGGucacGAGGcAGc 3′
|||||| |||| ||
3′ uucugCCCUCCc---CUCC-UCac 5′</td></tr>
<tr><td rowspan="3">ET-1</td><td>Human</td><td>-22.5</td><td>5′ uGACGa-AGGuccuaaGGGAGUGu 3′
|||| ||| |||||||
3′ uCUGCccUCCuc----UCCUCACu 5′</td></tr>
<tr><td>Mouse</td><td>-23.9</td><td>5′ cuguagaaAGuccuuaGGGAGUGu 3′
|| |||||||
3′ uucugcccUCccc---UCCUCACu 5′</td></tr>
<tr><td>Rat</td><td>-25.2</td><td>5′ uguagaaaguccuuagGGAGUGu 3′
||||||
3′ uguucugcccuccccuCCUCAC 5′</td></tr>
</table>

miR-483-5p

<table>
<tr><th>Name</th><th>Species</th><th>MFE</th><th>Site</th></tr>
<tr><td>TGF-β</td><td>Human</td><td>-22.9</td><td>5′ CUCCCa------CUCCC-UCUc 3′
||||| ||||| |||
3′ GAGGGaagaaagGAGGGcAGAa 5′</td></tr>
<tr><td></td><td>Mouse</td><td>-20.1</td><td>5′ gCCCggCcCcgCcCCCGcCc 3′
||| | | | |||| |
3′ aGGGaaGaGaaGaGGGCaGaa 5′</td></tr>
<tr><td></td><td>Rat</td><td>-18.7</td><td>5′ cCCCgcccgcagugCCCGgggCUg 3′
||| |||| ||
3′ aGGGaagagaaga-GGGCa--GAa 5′</td></tr>
<tr><td>TGFBR2</td><td>Human</td><td>-26.3</td><td>5′ cuuuccucaugguuaCCGUUc 3′
|||||
3′ gagggaagaaaggagGGCAGaa 5′</td></tr>
<tr><td></td><td>Mouse</td><td>-24.7</td><td>5′ aCCCUcCUUUUC-CUCGaUCa 3′
|||| |||||| |||| ||
3′ aGGGAaGAGAAGaGGGC-AGaa 5′</td></tr>
<tr><td></td><td>Rat</td><td>-17.1</td><td>5′ CUCCggaag-----UCCGUCc 3′
|||| ||||||
3′ GAGGgaagagaagaGGGCAGaa 5′</td></tr>
<tr><td>IL-1β</td><td>Human</td><td>-26.7</td><td>5′ gCCUggaCUUUCCUgUUGUCUa 3′
||| ||||||| ||||||
3′ aGGGaa-GAAAGGAgGGCAGAa 5′</td></tr>
<tr><td></td><td>Mouse</td><td>-16.3</td><td>5′ UCUCUagaacagaacCUagCUGUCa 3′
||||| || |||||
3′ AGGGAagagaa----GAg-GGCAGaa 5′</td></tr>
<tr><td></td><td>Rat</td><td>-22.4</td><td>5′ gCCUUUgUCcUCUgCCaaGUCa 3′
||||| || ||| || |||
3′ aGGGAAgAGaAGAgGG--CAGaa 5′</td></tr>
<tr><td>ET-1</td><td>Human</td><td>-22.5</td><td>5′ aCCUUCgggg--CCUGUCUg 3′
||||| |||||||
3′ gGGAAGaaaggaGGGCAGAa 5′</td></tr>
<tr><td></td><td>Mouse</td><td>-22.7</td><td>5′ uUCCCUag------CCUGUCUg 3′
||||| |||||||
3′ gAGGGAagagaagaGGGCAGAa 5′</td></tr>
<tr><td></td><td>Rat</td><td>-29.4</td><td>5′ UCUCUUgcCUCUUCUUgCUGUCUg 3′
|||||| |||||||| ||||||
3′ AGGGAA--GAGAAGAG-GGCAGAa 5′</td></tr>
</table>

Appendix Table S2. Primers used for qPCR

Gene	Species	Sequence: 5'-3'
GADPH	Homo sapiens	Forward: ACCACAGTCCATGCCATCAC
	Rattus norvegicus	Reverse: TCCACCACCCTGTTGCTGTA
TGF-β	Homo sapiens	Forward: TACCATGCCAACTTCTGCCT
		Reverse: ACGATCATGTTGGACAGCTG
TGF-β	Rattus norvegicus	Forward: TCCTTGCCCTCTACAACCAA
		Reverse: CGCACGATCATGTTGGACAA
TGFBR2	Homo sapiens	Forward: AGAAATTCCCAGCTTCTGGC
		Reverse: TCTGTTCTTTGGTGAGAGGG
TGFBR2	Rattus norvegicus	Forward: AGAAATTCCCAGCTTCTGGC
		Reverse: TCTGTTCTTTGGTGAGAGGG
β-catenin	Homo sapiens	Forward: GAACCCCTTGGATATCGCCA
		Reverse: AGGTCAGTATCAAACCAGGC
β-catenin	Rattus norvegicus	Forward: TTGGATATCGCCAGGACGAT
		Reverse: CCAGCTGATTGCTATCACCT
CTGF	Homo sapiens	Forward: TTACCAATGACAACGCCTCC
		Reverse: GTACGGATGCACTTTTTGCC
CTGF	Rattus norvegicus	Forward: TGCACCAGTGTGAAGACCTA
		Reverse: TGAGTTCGTGTCCCTTACTC
IL-1β	Homo sapiens	Forward: GATAAGCCCACTCTACAGCT
		Reverse: AGTCAGTTATATCCTGGCCG
IL-1β	Rattus norvegicus	Forward: GAAGATGGAAAAGCGGTTTG
		Reverse: AGGAAGACACGGGTTCCATG
ET-1	Homo sapiens	Forward: GGGCTGAAGACATTATGGAG
		Reverse: CGAAGGTCTGTCACCAATGT
ET-1	Rattus norvegicus	Forward: AAAACCCTGTCCCAAGCTGG
		Reverse: GGCTCTGTAGTCAATGTGCT
miR-483-3p-RT	Mus musculus	5'-CTCAACTGGTGTCGTGGAGTCG
		GCAATTCAGTTGAGAAGACGGG-3'
miR-483-3p	Mus musculus	Forward: CACTCCAGCTGGGATCACTCCTCCCCTCC
		Reverse: CTGGTGTCGTGGAGTCGG
miR-483-5p-RT	Mus musculus	5'-CTCAACTGGTGTCGTGGAGTCG
		GCAATTCAGTTGAGCTCCCTTC-3'
miR-483-5p	Mus musculus	Forward: CACTCCAGCTGGGAAAGACGGGAGAAGAGA
		Reverse: CTGGTGTCGTGGAGTCGG
miR-483-3p-RT	Rattus norvegicus	5'-CTCAACTGGTGTCGTGGAGTCG
		GCAATTCAGTTGAGACAAGACG-3'
miR-483-3p	Rattus norvegicus	Forward: CACTCCAGCTGGGACACTCCTCCCCTCCCG
		Reverse: CTGGTGTCGTGGAGTCGG
miR-483-5p-RT	Rattus norvegicus	5'-CTCAACTGGTGTCGTGGAGTCG
		GCAATTCAGTTGAGCTCCCTTC-3'
miR-483-5p	Rattus norvegicus	Forward: CACTCCAGCTGGGAAAGACGGGAGAAGAGA
		Reverse: CTGGTGTCGTGGAGTCGG

Appendix Table S3

Figure	Panel	p-value
1	A	miR-483-3p, P<0.0001; miR-483-5p, P<0.0001
1	B	miR-483-3p, P<0.0001; miR-483-5p, P<0.0001
1	C	miR-483-3p: Low vs. HC, P=0.0608; Inter & High vs. HC, P<0.0001; Inter & High vs. Low, P=0.0132; miR-483-5p: Inter & High vs. HC, P<0.0001; Inter & High vs. Low, P=0.093
1	D	ET-1, P=0.0229; IL-6, P=0.006
2	A	miR-483-3p, P<0.0001; miR-483-5p, P=0.0012
3	B	miR-483-3p, P=0.0267; miR-483-5p, P=0.0267
3	C	TGF-β, P=0.0147; TGFBR2, P=0.017; β-catenin, P=0.049; CTGF, P=0.037; IL-1β, P=0.011; ET-1, P=0.017
3	D	TGF-β, P=0.0063; TGFBR2, P=0.0041; β-catenin, P=0.0278; CTGF, P=0.0018; IL-1β, P=0.0011; ET-1, P=0.0078
3	E	Luc-TGF-β-WT, P=0.022; Luc-TGFBR2-WT, P=0.0331; Luc-IL-1β-WT, P=0.0072; Luc-ET-1-WT, P=0.043
3	F	Ago1-miR-483-3p, P=0.0449; Ago1-miR-483-5p, P=0.0187; Ago2-miR-483-3p, P=0.0093; Ago2-miR-483-5p, P=0.0049
3	G	Ago1-TGF-β, P=0.0487; Ago1-β-catenin, P=0.0433; Ago1-CTGF, P=0.0491; Ago1-ET-1, P=0.0234; Ago2-TGF-β, P=0.0226; Ago2-TGFBR2, P=0.0398; Ago2-β-catenin, P=0.0489; Ago2-CTGF, P=0.0402; Ago2-IL-1β, P=0.0424
4	A	miR-483-3p, P=0.0112; miR-483-5p, P=0.0141
4	B	miR-483-3p, P=0.0138; miR-483-5p, P=0.0322
4	C	WT+hypoxia vs. WT+normoxia, P=0.0239; Tg+hypoxia vs. WT+normoxia, P=0.0047
4	D	6 h: Tg+normoxia vs. WT+normoxia, P=0.0486; WT+hypoxia vs. WT+normoxia, P=0.0253; Tg+hypoxia vs. WT+normoxia, P=0.0424;<br>12 h: Tg+normoxia vs. WT+normoxia, P=0.0481; WT+hypoxia vs. WT+normoxia, P=0.0271; Tg+hypoxia vs. WT+normoxia, P=0.0273
4	E	mRNA: TGF-β: Tg+saline vs. WT+saline, P=0.0008; WT+MCT vs. WT+saline, P=0.0072; Tg+MCT vs. WT+MCT, P=0.0342; TGFBR2: Tg+saline vs. WT+saline, P=0.0373; WT+MCT vs. WT+saline, P=0.0151; Tg+MCT vs. WT+MCT, P=0.0747; β-catenin: Tg+saline vs. WT+saline, P=0.0031; WT+MCT vs. WT+saline, P=0.0037; Tg+MCT vs. WT+MCT, P=0.0051; CTGF: Tg+saline vs. WT+saline, P=0.0612; WT+MCT vs. WT+saline, P<0.0001; Tg+MCT vs. WT+MCT, P=0.0001; IL-1β: WT+MCT vs. WT+saline, P=0.0022; Tg+MCT vs. WT+MCT, P=0.002; ET-1: Tg+saline vs. WT+saline, P=0.0036; WT+MCT vs. WT+saline, P=0.0014; Tg+MCT vs. WT+MCT, P=0.0365<br>Protein: TGF-β: Tg+saline vs. WT+saline, P=0.0037; WT+MCT vs. WT+saline, P=0.0067; Tg+MCT vs. WT+MCT, P=0.0083; TGFBR2: Tg+saline vs. WT+saline, P=0.0007; WT+MCT vs. WT+saline, P=0.0005; Tg+MCT vs. WT+MCT, P=0.004; β-catenin: Tg+saline vs. WT+saline, P=0.063; WT+MCT vs. WT+saline, P=0.0173; Tg+MCT vs. WT+MCT, P=0.0024; CTGF: Tg+saline vs. WT+saline, P=0.025;

		WT+MCT vs. WT+saline, P=0.0233; Tg+MCT vs. WT+MCT, P=0.0048; IL-1β: Tg+saline vs. WT+saline, P=0.0026; WT+MCT vs. WT+saline, P=0.0071; Tg+MCT vs. WT+MCT, P=0.0123; ET-1: Tg+saline vs. WT+saline, P=0.0386; WT+MCT vs. WT+saline, P=0.0182; Tg+MCT vs. WT+MCT, P=0.0419
4	F	Sera: miR-483-3p: Tg+saline vs. WT+saline, P=0.0234; WT+MCT vs. WT+saline, P=0.0446; Tg+MCT vs. WT+MCT, P=0.0422; miR-483-5p: Tg+saline vs. WT+saline, P=0.0644; WT+MCT vs. WT+saline, P=0.0206; Tg+MCT vs. WT+MCT, P=0.0325<br>Lung: miR-483-3p: Tg+saline vs. WT+saline, P=0.0044; Tg+MCT vs. WT+MCT, P=0.0102; miR-483-5p: Tg+saline vs. WT+saline, P=0.0195; Tg+MCT vs. WT+MCT, P=0.0197
4	G	Ago1: miR-483-3p: Tg+saline vs. WT+saline, P=0.0378; WT+MCT vs. WT+saline, P=0.0498; Tg+MCT vs. WT+MCT, P=0.0085; miR-483-5p: Tg+saline vs. WT+saline, P=0.0421; WT+MCT vs. WT+saline, P=0.0247; Tg+MCT vs. WT+MCT, P=0.0398<br>Ago2: miR-483-3p: Tg+saline vs. WT+saline, P=0.0117; Tg+MCT vs. WT+MCT, P=0.0353; miR-483-5p: Tg+saline vs. WT+saline, P=0.036; WT+MCT vs. WT+saline, P=0.0494; Tg+MCT vs. WT+MCT, P=0.02
4	H	Ago1: TGF-β: WT+MCT vs. WT+saline, P=0.0196; TGFBR2: WT+MCT vs. WT+saline, P=0.0776; β-catenin: Tg+saline vs. WT+saline, P=0.0606; WT+MCT vs. WT+saline, P=0.0086; Tg+MCT vs. WT+MCT, P=0.069; CTGF: Tg+saline vs. WT+saline, P=0.0085; Tg+MCT vs. WT+MCT, P=0.0039; IL-1β: WT+MCT vs. WT+saline, P=0.0689; Tg+MCT vs. WT+MCT, P=0.0317; ET-1: Tg+saline vs. WT+saline, P=0.047; WT+MCT vs. WT+saline, P=0.0137; Tg+MCT vs. WT+MCT, P=0.0799<br>Ago2: TGF-β: Tg+saline vs. WT+saline, P=0.016; WT+MCT vs. WT+saline, P=0.0035; TGFBR2: WT+MCT vs. WT+saline, P=0.0588; Tg+MCT vs. WT+MCT, P=0.0083; β-catenin: Tg+saline vs. WT+saline, P=0.0717; WT+MCT vs. WT+saline, P=0.0176; Tg+MCT vs. WT+MCT, P=0.0393; CTGF: Tg+saline vs. WT+saline, P=0.0107; WT+MCT vs. WT+saline, P=0.0295; Tg+MCT vs. WT+MCT, P=0.0053; IL-1β: WT+MCT vs. WT+saline, P=0.0856; Tg+MCT vs. WT+MCT, P=0.0379; ET-1: Tg+saline vs. WT+saline, P=0.019; WT+MCT vs. WT+saline, P=0.0001; Tg+MCT vs. WT+MCT, P=0.0532
5	A	WT+MCT vs. WT+saline, P=0.0006; Tg+MCT vs. WT+MCT, P=0.0006
5	B	WT+MCT vs. WT+saline, P=0.0043; Tg+MCT vs. WT+MCT, P=0.0024
5	C	WT+MCT vs. WT+saline, P<0.0001; Tg+MCT vs. WT+MCT, P<0.0001
5	D	WT+MCT vs. WT+saline, P=0.0004; Tg+MCT vs. WT+MCT, P=0.0014
5	E	WT+SU/hypoxia vs. WT+normoxia, P=0.0156; Tg+SU/hypoxia vs. WT+SU/hypoxia, P=0.0005
5	E	WT+SU/hypoxia vs. WT+normoxia, P<0.0001; Tg+SU/hypoxia vs. WT+SU/hypoxia, P=0.06
6	B	miR-483-3p: M+GFP vs. S+GFP, P=0.0476; M+483 vs. M+GFP, P=0.0127<br>miR-483-5p: M+GFP vs. S+GFP, P=0.0382; M+483 vs. M+GFP, P=0.0018
6	C	miR-483-3p: M+GFP vs. S+GFP, P=0.0163; M+483 vs. M+GFP, P=0.0121<br>miR-483-5p: M+GFP vs. S+GFP, P=0.0343; M+483 vs. M+GFP, P=0.04
6	D	miR-483-3p: M+GFP vs. S+GFP, P=0.0163; M+483 vs. M+GFP, P=0.0121

		mRNA: TGF-β: M+GFP vs. S+GFP, P=0.0406; M+483 vs. M+GFP, P=0.0397; TGFBR2: M+GFP vs. S+GFP, P=0.0121; M+483 vs. M+GFP, P=0.0077; β-catenin: M+GFP vs. S+GFP, P=0.0417; M+483 vs. M+GFP, P=0.0474; CTGF: M+GFP vs. S+GFP, P=0.0315; M+483 vs. M+GFP, P=0.236; IL-1β: M+GFP vs. S+GFP, P=0.0673; M+483 vs. M+GFP, P=0.132; ET-1: M+GFP vs. S+GFP, P=0.0243; M+483 vs. M+GFP, P=0.0043
		Protein: TGF-β: M+GFP vs. S+GFP, P=0.0055; M+483 vs. M+GFP, P=0.0154; TGFBR2: M+GFP vs. S+GFP, P=0.0007; M+483 vs. M+GFP, P=0.0007; β-catenin: M+GFP vs. S+GFP, P=0.013; M+483 vs. M+GFP, P=0.0143; CTGF: M+GFP vs. S+GFP, P=0.0175; M+483 vs. M+GFP, P=0.0245; IL-1β: M+GFP vs. S+GFP, P=0.0086; M+483 vs. M+GFP, P=0.0481; ET-1: M+GFP vs. S+GFP, P=0.0034; M+483 vs. M+GFP, P=0.0369
6	E	M+GFP vs. S+GFP, P<0.0001; M+483 vs. M+GFP, P=0.0014
6	F	M+GFP vs. S+GFP, P<0.0001; M+483 vs. M+GFP, P=0.01
6	H	M+GFP vs. S+GFP, P<0.0001; M+483 vs. M+GFP, P<0.0001
6	I	M+GFP vs. S+GFP, P<0.0001; M+483 vs. M+GFP, P<0.0001
6	J	M+483 vs. M+GFP, P=0.058
Appendix Fig. S1	A	P<0.0001
Appendix Fig. S1	B	P<0.0001
Appendix Fig. S1	C	P<0.0001
Appendix Fig. S1	D	P<0.0001
Appendix Fig. S2	A	miR-483-3p, P=0.0175; miR-483-5p, P=0.0218
Appendix Fig. S2	B	TGF-β, P=0.008; TGFBR2, P=0.0453; β-catenin, P=0.0427; CTGF, P=0.0021; IL-1β, P=0.0377; ET-1, P=0.0193
Appendix Fig. S2	C	TGF-β, P=0.0006; TGFBR2, P=0.0109; β-catenin, P=0.0192; CTGF, P=0.0286; IL-1β, P=0.0064; ET-1, P=0.0196
Appendix Fig. S3	A	miR-483-3p, P=0.0122; miR-483-5p, P=0.0026
Appendix Fig. S4	A	Sera: miR-483-3p, P=0.0242; miR-483-5p, P=0.0431<br>Lung: miR-483-3p, P=0.008; miR-483-5p, P=0.0201
Appendix Fig. S4	B	TGF-β, P=0.0194; TGFBR2, P=0.054; β-catenin, P=0.0513; CTGF, P=0.0444; IL-1β, P=0.0086; ET-1, P=0.0005
Appendix Fig. S4	C	TGF-β, P=0.0298; TGFBR2, P=0.0344; β-catenin, P=0.0049; CTGF, P=0.0332; IL-1β, P=0.0259; ET-1, P=0.0105
Appendix Fig. S4	D	P=0.0216
Appendix Fig. S4	E	P=0.0337
Appendix Fig. S4	F	P=0.0007
Appendix Fig. S5	A	Sera: miR-483-3p: Tg+normoxia vs. WT+normoxia, P=0.0295; WT+SU/hypoxia vs. WT+normoxia, P=0.023; Tg+SU/hypoxia vs. WT+SU/hypoxia, P=0.0054; miR-483-5p: Tg+normoxia vs. WT+normoxia, P=0.0051; WT+SU/hypoxia vs. WT+normoxia, P=0.0212; Tg+SU/hypoxia vs. WT+SU/hypoxia, P=0.0002<br>Lung: miR-483-3p: Tg+normoxia vs. WT+normoxia, P=0.0395; WT+SU/hypoxia vs. WT+normoxia, P=0.0574; Tg+SU/hypoxia vs. WT+SU/hypoxia, P=0.0003; miR-483-5p: Tg+normoxia vs. WT+normoxia, P=0.0054; WT+SU/hypoxia vs. WT+normoxia, P=0.0408; Tg+SU/hypoxia vs. WT+SU/hypoxia, P=0.011
Appendix Fig. S5	C	mRNA: TGF-β: WT+SU/hypoxia vs. WT+normoxia, P=0.0107; Tg+SU/hypoxia vs. WT+SU/hypoxia, P=0.0136; TGFBR2: WT+SU/hypoxia vs. WT+normoxia, P=0.0078;

	Tg+SU/hypoxia vs. WT+SU/hypoxia, P=0.0094; β-catenin: WT+SU/hypoxia vs. WT+normoxia, P=0.0457; Tg+SU/hypoxia vs. WT+SU/hypoxia, P=0.0426; CTGF: WT+SU/hypoxia vs. WT+normoxia, P=0.0309; Tg+SU/hypoxia vs. WT+SU/hypoxia, P=0.0262; IL-1β: WT+SU/hypoxia vs. WT+normoxia, P=0.0303; Tg+SU/hypoxia vs. WT+SU/hypoxia, P=0.048; ET-1: WT+SU/hypoxia vs. WT+normoxia, P=0.0458; Tg+SU/hypoxia vs. WT+SU/hypoxia, P=0.0295<br>Protein: TGF-β: WT+SU/hypoxia vs. WT+normoxia, P=0.0247; Tg+SU/hypoxia vs. WT+SU/hypoxia, P=0.0429; TGFBR2: WT+SU/hypoxia vs. WT+normoxia, P=0.0399; Tg+SU/hypoxia vs. WT+SU/hypoxia, P=0.0362; β-catenin: Tg+normoxia vs. WT+normoxia, P=0.0046, WT+SU/hypoxia vs. WT+normoxia, P=0.0423; Tg+SU/hypoxia vs. WT+SU/hypoxia, P=0.0075; CTGF: Tg+normoxia vs. WT+normoxia, P=0.0037; WT+SU/hypoxia vs. WT+normoxia, P=0.0302; Tg+SU/hypoxia vs. WT+SU/hypoxia, P=0.0032; IL-1β: WT+SU/hypoxia vs. WT+normoxia, P=0.032; Tg+SU/hypoxia vs. WT+SU/hypoxia, P=0.0138; ET-1: WT+SU/hypoxia vs. WT+normoxia, P=0.034; Tg+SU/hypoxia vs. WT+SU/hypoxia, P=0.022
Appendix Fig. S7	miR-483-3p, P=0.0236; miR-483-5p, P=0.0495
Appendix Fig. S9	miR-483-3p, P=0.0025; miR-483-5p, P=0.0126
Appendix Fig. S10A	miR-483-3p, P=0.015; miR-483-5p, P=0.076
Appendix Fig. S10B	miR-483-3p, P=0.0146; miR-483-5p, P=0.0221
