# Supplementary material for: MicroRNA‐483 amelioration of experimental pulmonary hypertension
Source: EMBO Mol Med. 2020 Apr 23;12(5):e11303. doi: 10.15252/emmm.201911303 (PMC7207157; doi:10.15252/emmm.201911303)
Supplement: Supplementary file 2 — Source Data for Appendix [file EMMM-12-e11303-s008.zip › Source_Data_for_Appendix_Figures/Source_data_for_Appendix_Fig.S3.pdf]

Fig.S3C

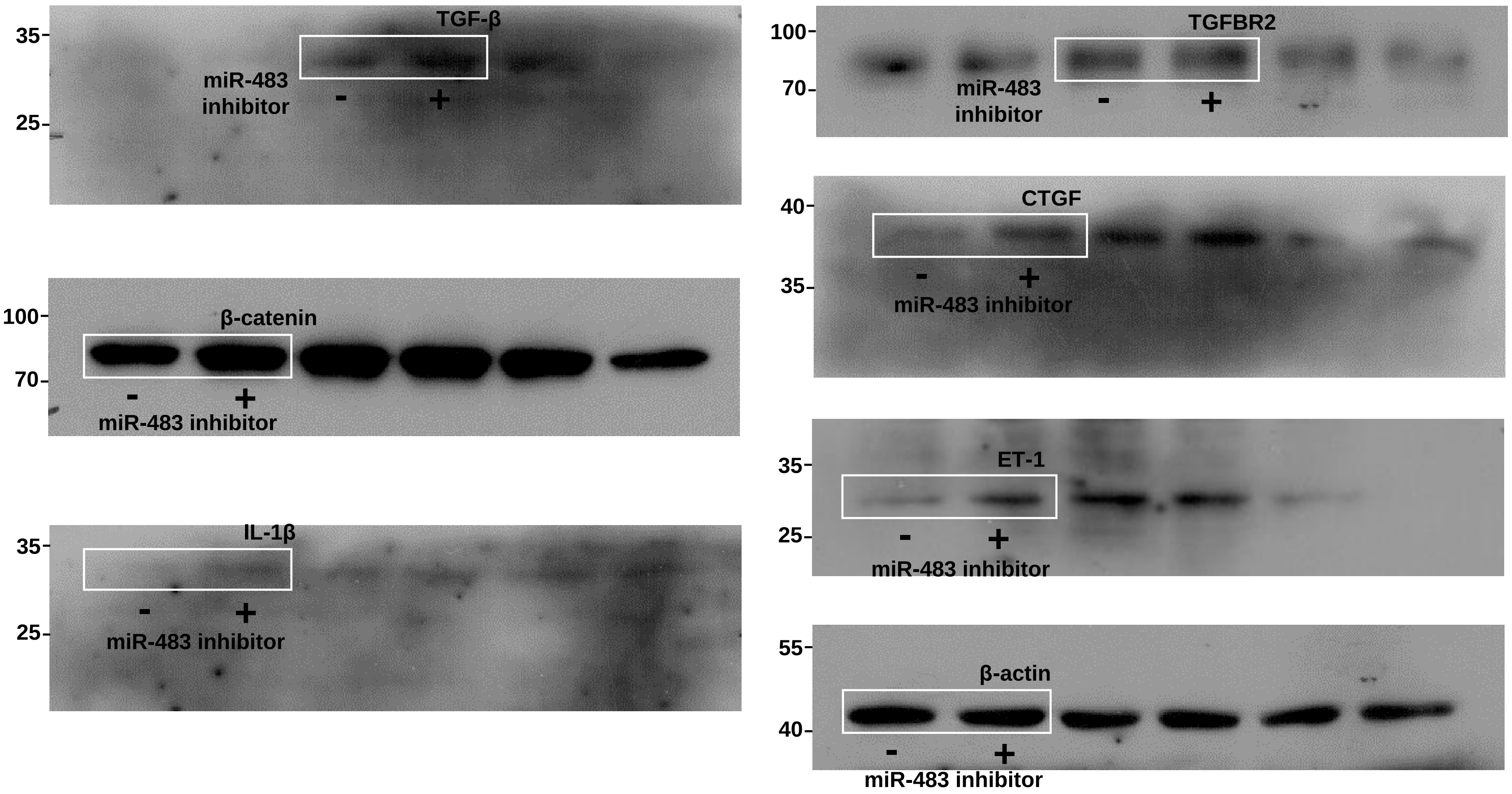

TGF-β
35-
25-
miR-483
inhibitor
-
+
TGFBR2
100-
70-
miR-483
inhibitor
-
+
CTGF
40-
35-
-
+
miR-483 inhibitor
β-catenin
100-
70-
-
+
miR-483 inhibitor
ET-1
35-
25-
-
+
miR-483 inhibitor
IL-1β
35-
25-
-
+
miR-483 inhibitor
β-actin
55-
40-
-
+
miR-483 inhibitor

Fig.S3A

	Scramble			miR483 inhibitor		
miR-483-3p	0.818619	1.132016	1.049868	0.388347	0.53966	0.664908
miR-483-5p	0.965059	0.809289	1.226031	0.459332	0.065773	0.359713

Fig.S3B

	Scramble			miR483 inhibitor		
TGF-β	0.823502	1.218483	0.957952	2.830718	1.695871	2.426547
TGFBR2	0.644727	1.753561	0.601716	2.378176	2.321624	2.375737
β-catenin	0.303191	2.240119	0.456689	2.815602	3.892416	4.008903
CTGF	0.933993	0.777063	1.288946	2.121873	2.147734	1.581715
IL-1β	0.85172	0.433064	1.71406	1.232785	2.227685	1.389742
ET-1	0.918061	0.958953	1.122985	1.96745	2.683421	2.688548

Fig.S3C

	Scramble			miR483 inhibitor		
TGF-β	1	1	1	2.173444	2.250026	2.156395
TGFBR2	1	1	1	1.794407	1.61645	1.565767
β-catenin	1	1	1	1.779577	2.154905	1.770404
CTGF	1	1	1	1.85295	2.087906	2.535527
IL-1β	1	1	1	1.723022	1.913902	1.724313
ET-1	1	1	1	2.16204	1.81862	1.743205

All data were fold changes, normalized to "scramble".
