# Supplementary material for: MicroRNA‐483 amelioration of experimental pulmonary hypertension
Source: EMBO Mol Med. 2020 Apr 23;12(5):e11303. doi: 10.15252/emmm.201911303 (PMC7207157; doi:10.15252/emmm.201911303)
Supplement: Supplementary file 2 — Source Data for Appendix [file EMMM-12-e11303-s008.zip › Source_Data_for_Appendix_Figures/Source_data_for_Appendix_Fig.S6.pdf]

Fig.S6B

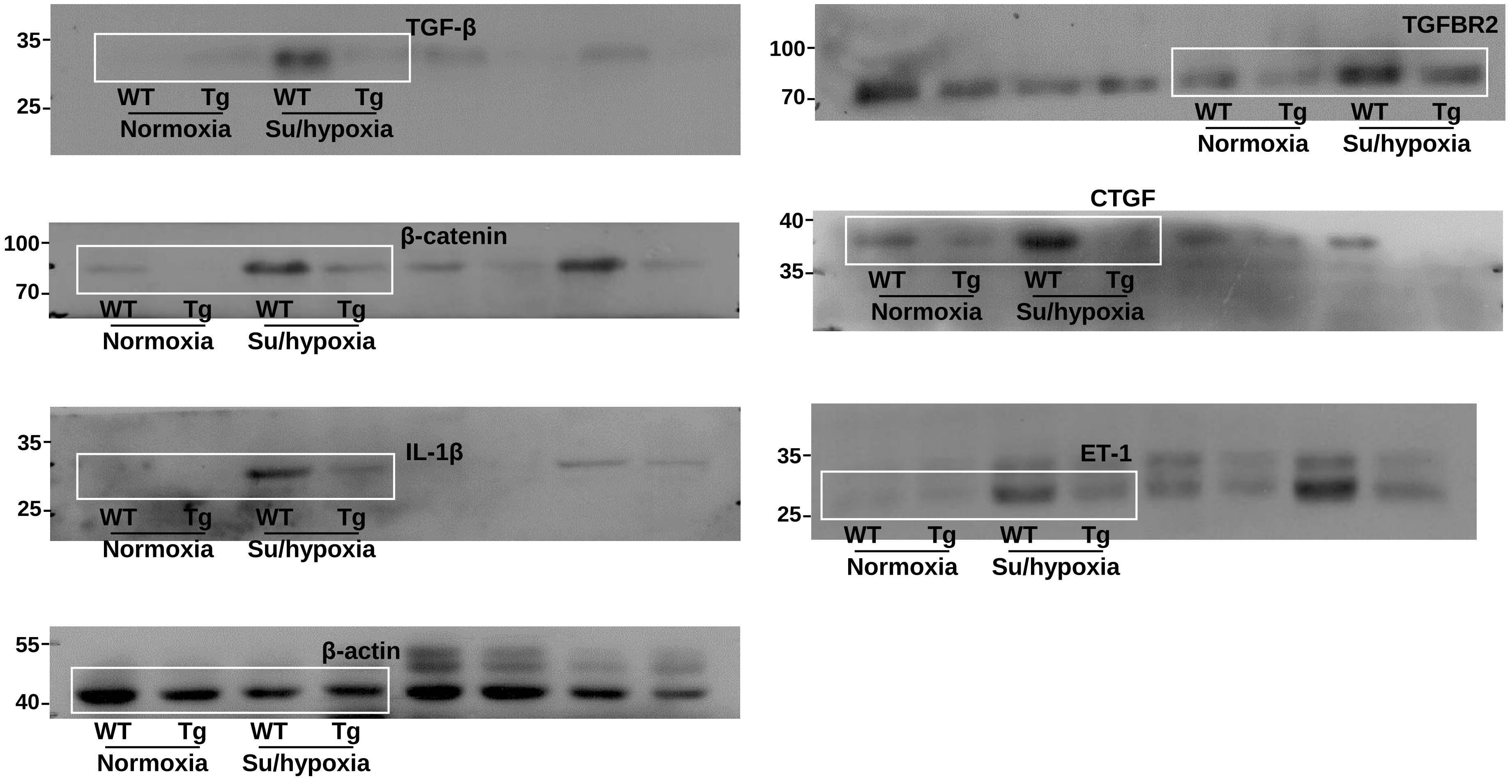
TGF-β
35
25
WT Tg WT Tg
Normoxia Su/hypoxia
TGFBR2
100
70
WT Tg WT Tg
Normoxia Su/hypoxia
β-catenin
100
70
WT Tg WT Tg
Normoxia Su/hypoxia
CTGF
40
35
WT Tg WT Tg
Normoxia Su/hypoxia
IL-1β
35
25
WT Tg WT Tg
Normoxia Su/hypoxia
ET-1
35
25
WT Tg WT Tg
Normoxia Su/hypoxia
β-actin
55
40
WT Tg WT Tg
Normoxia Su/hypoxia

Fig.S6A

	WT+normoxia			Tg+normoxia			WT+Su/hypoxia			Tg+Su/hypoxia		
sera-miR-483-3p	0.92539	0.94707	1.12754	2.72962	1.90939	2.5096	0.35933	0.57064	0.47217	2.23151	2.06209	2.35523
sera-miR-483-5p	1.05731	1.12009	0.82259	2.8284	3.12679	2.3862	0.62948	0.43528	0.34372	2.67984	2.54562	2.35903
lung-miR-483-3p	1.42741	0.77838	0.79433	2.26811	2.53845	2.07724	0.28507	0.29081	0.10545	2.19231	2.26801	2.12488
lung-miR-483-5p	1.04828	1.17887	0.77286	2.90913	2.64786	2.60982	0.29569	0.58909	0.4246	2.09908	2.23663	2.74441

Fig.S6C

mRNA	WT+normoxia			Tg+normoxia			WT+Su/hypoxia			Tg+Su/hypoxia		
TGF-β	0.82409	0.80399	1.37207	2.48271	0.57318	0.73833	5.17779	5.69479	4.76212	1.83613	1.19982	1.66133
TGFBR2	0.76124	1.12199	1.11777	1.79083	0.67421	0.55673	3.02573	3.86464	3.15229	1.97659	2.38552	1.86736
β-catenin	0.94067	0.97748	1.08481	0.91915	1.12793	0.98844	2.20269	2.55853	1.90749	1.16487	1.68587	1.33609
CTGF	1.14542	0.79959	1.05498	0.55865	1.38853	1.06815	9.20824	6.82106	4.2291	1.42826	1.35705	1.34212
IL-1β	1.09768	1.00192	0.9004	1.30353	0.58186	1.28503	5.76615	3.98007	3.6042	1.23543	0.66094	1.6475
ET-1	0.48032	1.43359	1.0861	0.42849	1.66867	0.63552	2.09341	2.21061	2.08297	1.17581	1.51304	0.8038
protein	WT+normoxia			Tg+normoxia			WT+Su/hypoxia			Tg+Su/hypoxia		
TGF-β	1	1	1	1.08652	0.92836	1.2104	1.86327	2.3644	1.87161	1.19321	0.90085	0.79649
TGFBR2	1	1	1	0.65877	0.72301	0.90605	1.84032	2.50489	1.8526	1.01644	0.84977	0.64806
β-catenin	1	1	1	0.71539	0.65153	0.69781	2.217	2.21311	3.00417	0.87048	0.57564	1.48465
CTGF	1	1	1	0.5604	0.6536	0.60239	1.78437	2.30054	1.66588	0.48762	0.81478	0.57028
IL-1β	1	1	1	0.68893	0.82399	1.0142	2.00057	2.70578	2.04004	0.80978	1.37385	1.16256
ET-1	1	1	1	0.74629	0.62205	0.2186	1.72768	2.01321	1.52098	0.72985	0.60455	0.65956

All data were fold changes, normalized to "WT+normoxia".
