# Supplementary material for: MicroRNA‐483 amelioration of experimental pulmonary hypertension
Source: EMBO Mol Med. 2020 Apr 23;12(5):e11303. doi: 10.15252/emmm.201911303 (PMC7207157; doi:10.15252/emmm.201911303)
Supplement: Supplementary file 2 — Source Data for Appendix [file EMMM-12-e11303-s008.zip › Source_Data_for_Appendix_Figures/Source_data_for_Appendix_Fig.S5.pdf]

**Fig.S5C**

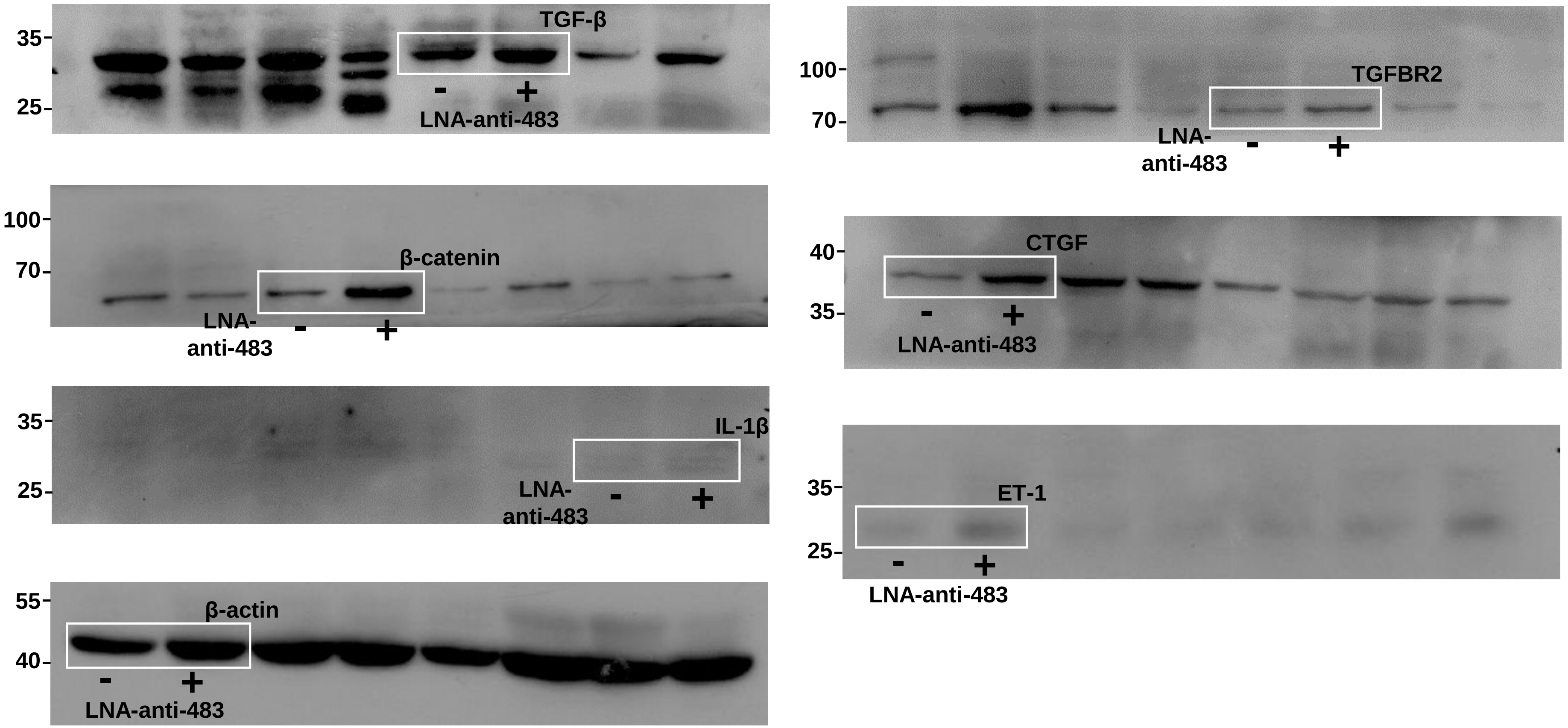

TGF-β
35
25
- +
LNA-anti-483
TGFBR2
100
70
LNA-anti-483
- +
β-catenin
100
70
LNA-anti-483
- +
CTGF
40
35
- +
LNA-anti-483
IL-1β
35
25
LNA-anti-483
- +
ET-1
35
25
- +
LNA-anti-483
β-actin
55
40
- +
LNA-anti-483

Fig.S5A

| | Scramble | | | LNA-anti-483 | | |
|---|---|---|---|---|---|---|
| sera-miR-483-3p | 0.967634 | 0.950483 | 1.081882 | 0.506293 | 0.438517 | 0.458939 |
| sera-miR-483-5p | 1.010452 | 0.848279 | 1.141271 | 0.543255 | 0.076781 | 0.421309 |
| lung-miR-483-3p | 0.677913 | 1.104261 | 1.217828 | 0.221002 | 0.322744 | 0.65087 |
| lung-miR-483-5p | 1.139103 | 1.245028 | 0.615891 | 0.15573 | 0.179409 | 0.130696 |

Fig.S5B

| | Scramble | | | LNA-anti-483 | | |
|---|---|---|---|---|---|---|
| TGF-β | 1.147459 | 1.077003 | 0.7755397 | 2.588565 | 2.732351 | 1.77541 |
| TGFBR2 | 1.078977 | 0.9159077 | 1.005152 | 3.386897 | 2.959358 | 1.928734 |
| β-catenin | 0.4053201 | 0.7232787 | 1.870759 | 3.56493 | 6.992041 | 5.599755 |
| CTGF | 1.172743 | 0.6731018 | 1.154123 | 1.718615 | 1.901643 | 1.930891 |
| IL-1β | 0.9793148 | 0.7586491 | 1.262037 | 2.93328 | 3.228277 | 3.096256 |
| ET-1 | 0.9310989 | 0.8361167 | 1.232781 | 1.869648 | 1.757965 | 2.223768 |

Fig.S5C

| | Scramble | | | LNA-anti-483 | | |
|---|---|---|---|---|---|---|
| TGF-β | 1 | 1 | 1 | 2.710092 | 2.643638 | 1.923599 |
| TGFBR2 | 1 | 1 | 1 | 1.433897 | 1.732288 | 1.873332 |
| β-catenin | 1 | 1 | 1 | 2.544523 | 2.347826 | 2.722014 |
| CTGF | 1 | 1 | 1 | 2.423034 | 2.01699 | 1.74054 |
| IL-1β | 1 | 1 | 1 | 2.104995 | 1.622681 | 2.04337 |
| ET-1 | 1 | 1 | 1 | 3.159048 | 2.820332 | 2.506295 |

Fig.S5D

| Scramble | LNA-anti-483 |
|---|---|
| 44.07 | 47.03 |
| 46.56 | 59.09 |
| 39.78 | 59.4 |
| 40.41 | 52.44 |
| 35.05 | 46.45 |
| 36.53 | 54.24 |

Fig.S5E

| Scramble | LNA-anti-483 |
|---|---|
| 0.586957 | 0.665487 |
| 0.481387 | 0.628945 |
| 0.40194 | 0.529994 |
| 0.480271 | 0.50887 |
| 0.396271 | 0.756493 |
| 0.58348 | 0.58735 |

Fig.S5F

| Scramble | LNA-anti-483 |
|---|---|
| 56.62343 | 87.36987 |
| 63.1758 | 79.55239 |
| 61.30084 | 77.0344 |
| 50.95713 | 79.80747 |
| 52.67557 | 71.32481 |
| 55.06002 | 70.69265 |

All data were fold changes, normalized to "scramble".
