# Supplementary material for: MicroRNA‐483 amelioration of experimental pulmonary hypertension
Source: EMBO Mol Med. 2020 Apr 23;12(5):e11303. doi: 10.15252/emmm.201911303 (PMC7207157; doi:10.15252/emmm.201911303)
Supplement: Supplementary file 6 — Source Data for Figure 3 [file EMMM-12-e11303-s004.pdf]

Fig.3D

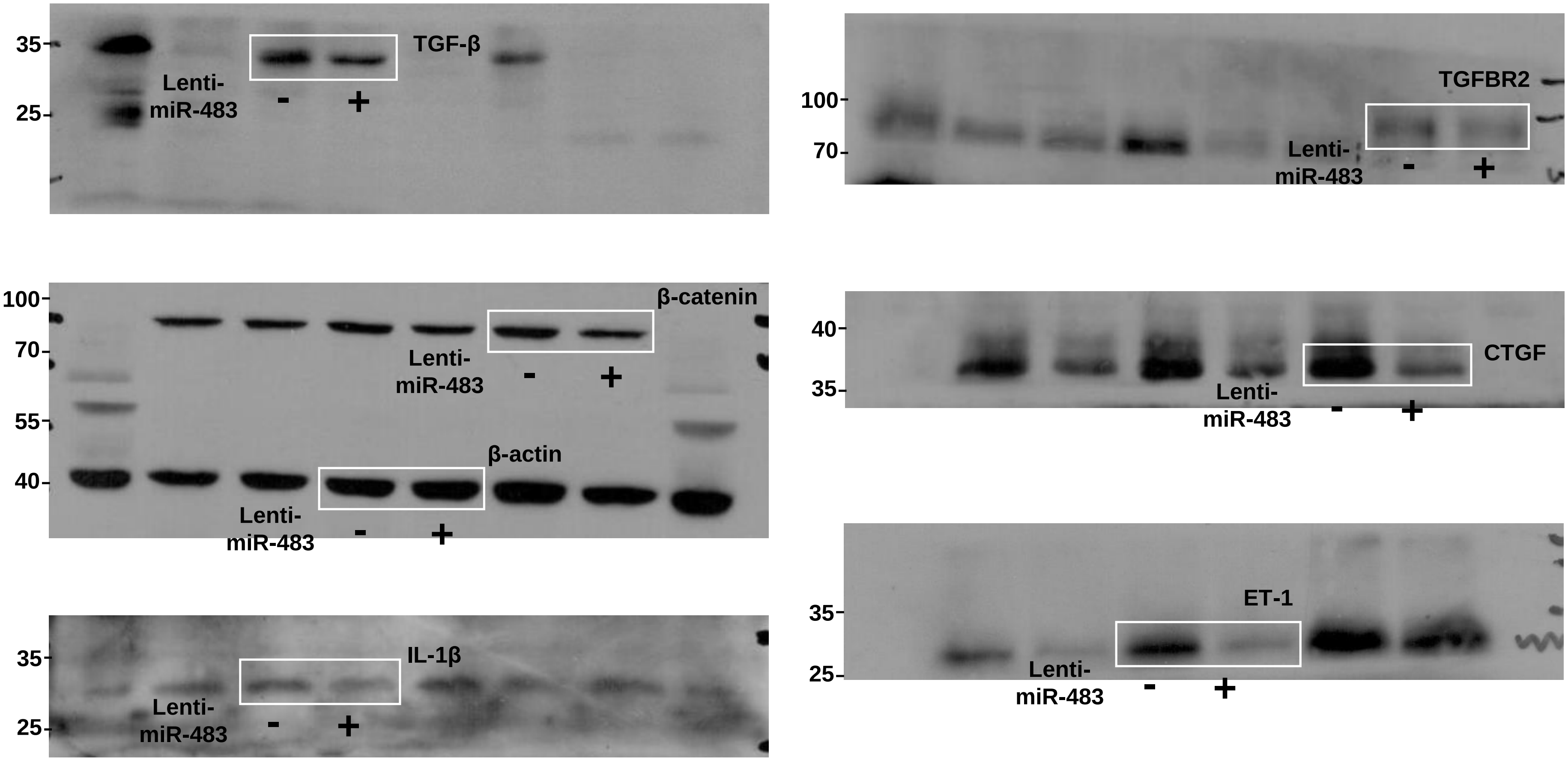

35
25
TGF-β
Lenti-miR-483
-
+
100
70
TGFBR2
Lenti-miR-483
-
+
100
70
55
40
β-catenin
Lenti-miR-483
-
+
β-actin
Lenti-miR-483
-
+
40
35
CTGF
Lenti-miR-483
-
+
35
25
IL-1β
Lenti-miR-483
-
+
35
25
ET-1
Lenti-miR-483
-
+

Fig.3B

	scramble			lenti-483		
miR-483-3p	1.16296	0.845456	0.99158	3.966321	5.727086	5.929051
miR-483-5p	1.111079	0.830448	1.058456	9.266094	5.321974	8.409159

Fig.3C

	scramble			lenti-483		
TGF-β	0.989196	0.935837	1.074994	0.2614	0.380068	0.216784
TGFBR2	1.094294	1	1.071773	0.610423	0.677307	0.565611
β-catenin	1.058593	1.166469	0.774936	0.72304	0.780326	0.674621
CTGF	1.231934	0.920778	0.847288	0.688212	0.594985	0.547498
IL-1β	1.495346	0.603104	0.90155	0.216206	0.273664	0.220749
ET-1	1.369483	0.854784	0.775733	0.684742	0.50126	0.703992

Fig.3D

	scramble			lenti-483		
TGF-β	1	1	1	0.522399	0.665313	0.604259
TGFBR2	1	1	1	0.336979	0.465386	0.426546
β-catenin	1	1	1	0.661908	0.763561	0.565042
CTGF	1	1	1	0.500979	0.550695	0.56649
IL-1β	1	1	1	0.376319	0.301595	0.333829
ET-1	1	1	1	0.303304	0.451481	0.468631

Fig.3E

	scramble			lenti-483		
Luc-TGF-β(WT)	1.03376	1.062669	0.903586	0.579409	0.566357	0.612042
Luc-TGF-β(mut)	1.166666	0.942459	0.890874	1.322892	1.139945	0.930259
Luc-TGFBR2(WT)	1.015276	0.927953	1.056755	0.652966	0.644375	0.524893
Luc-TGFBR2(mut)	0.992877	1.035935	0.971189	0.870312	0.863667	1.079262
Luc-IL1β(WT)	1.093692	0.954277	0.95207	0.767289	0.711962	0.654486
Luc-IL1β(mut)	1.039271	0.985932	0.974821	1.077521	1.159345	0.987603
Luc-ET1(WT)	1.042225	0.852954	1.104924	0.58278	0.59407	0.529522
Luc-ET1(mut)	1.080859	0.933516	0.9855	0.997354	0.861928	1.883404

Fig.3F

	scramble			lenti-483		
Ago1-miR-483-3p	1	1	1	3.1	2.86791	2.20381
Ago1-miR-483-5p	1	1	1	2.378414	2.928171	2.378414
Ago2-miR-483-3p	1	1	1	1.972465	2.694467	2.056228
Ago2-miR-483-5p	1	1	1	1.815038	1.60214	1.453972

Fig.3G

	scramble			lenti-483		
Ago1-TGF-β	1	1	1	2.281528	4.924578	2.86791
Ago1-TGFBR2	1	1	1	15.77972	2.989698	5.388934
Ago1-β-catenin	1	1	1	7.361501	3.271608	6.543217
Ago1-CTGF	1	1	1	2.297397	4.228072	2.713209
Ago1-IL-1β	1	1	1	4.500234	2.770219	2.928171
Ago1-ET-1	1	1	1	3.630077	3.917681	2.675855
Ago2-TGF-β	1	1	1	1.613284	1.60214	1.36604
Ago2-TGFBR2	1	1	1	2.20381	1.658639	2.411616
Ago2-β-catenin	1	1	1	22.3159	10.33882	8.514961
Ago2-CTGF	1	1	1	2.158457	1.433955	1.464086
Ago2-IL-1β	1	1	1	4.993322	2.907945	3.680751
Ago2-ET-1	1	1	1	1.658639	2.675855	2.73208

All data were fold changes, normalized to "scramble".
