# Supplementary material for: MicroRNA‐483 amelioration of experimental pulmonary hypertension
Source: EMBO Mol Med. 2020 Apr 23;12(5):e11303. doi: 10.15252/emmm.201911303 (PMC7207157; doi:10.15252/emmm.201911303)
Supplement: Supplementary file 7 — Source Data for Figure 4 [file EMMM-12-e11303-s005.pdf]

Fig.4E

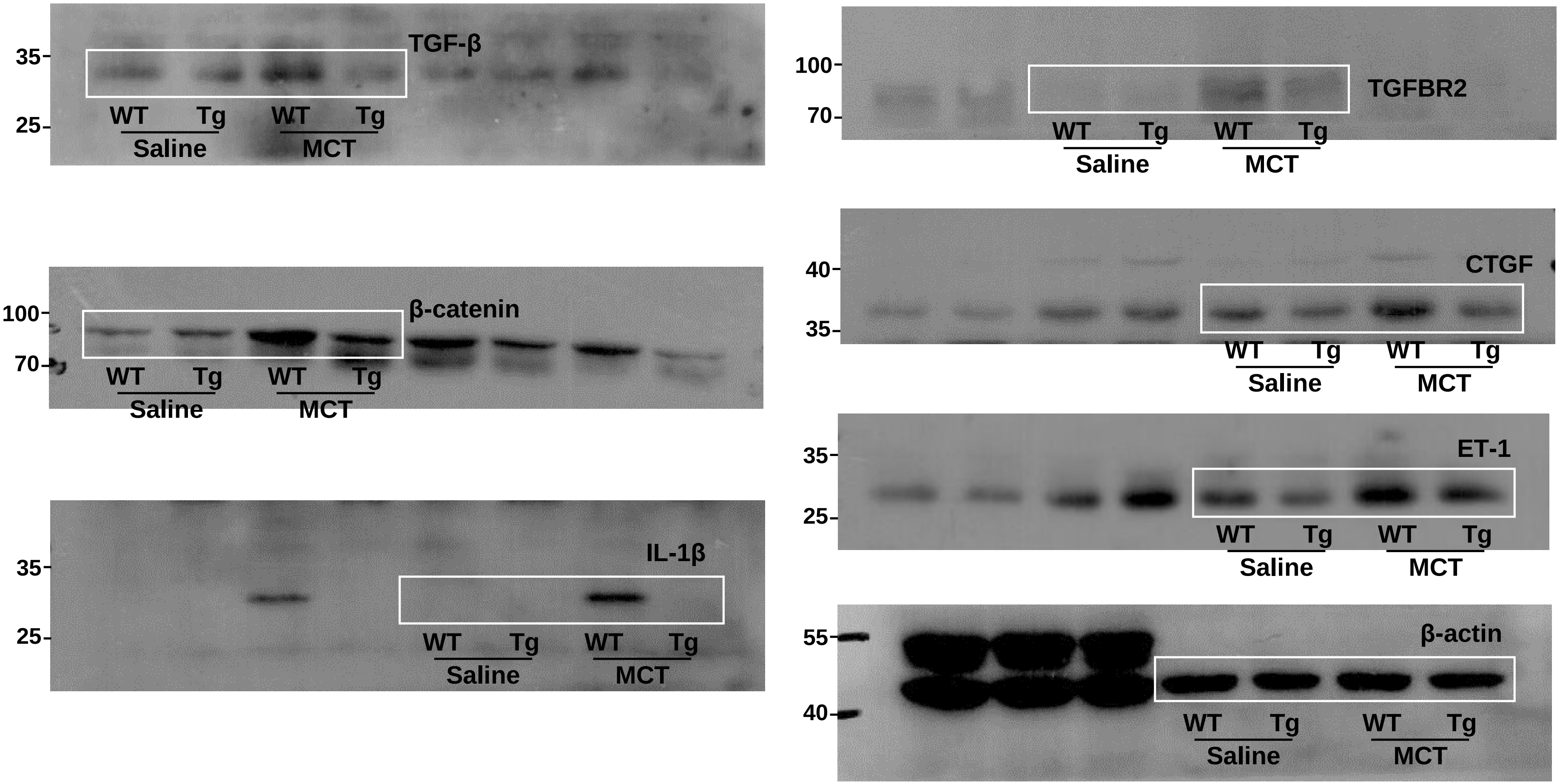

TGF-β
35
25
WT Tg WT Tg
Saline MCT
β-catenin
100
70
WT Tg WT Tg
Saline MCT
IL-1β
35
25
WT Tg WT Tg
Saline MCT
TGFBR2
100
70
WT Tg WT Tg
Saline MCT
CTGF
40
35
WT Tg WT Tg
Saline MCT
ET-1
35
25
WT Tg WT Tg
Saline MCT
β-actin
55
40
WT Tg WT Tg
Saline MCT

Fig.4A

	WT			Tg		
miR-483-3p	1.23672	0.57695	1.18634	2.96188	4.30649	4.27674
miR-483-5p	2.03614	0.83268	0.13359	6.84872	9.48623	8.72911

Fig.4B

	WT						Tg					
CD144-miR-483-3p	1.06614	1.15861	1.14266	0.8024	1.18295	0.64725	1.41656	1.3217	2.25385	2.62514	2.13227	1.63852
CD144-miR-483-5p	1.31982	0.81245	1.70568	0.65535	0.89524	0.61147	7.11244	3.27239	2.11454	2.05672	1.98666	3.1829

Fig.4C

	WT+normoxia			Tg+normoxia			WT+hypoxia			Tg+hypoxia		
S phase cells (%)	13.36	8.4	6.01	6.01	7.87	9.32	28.34	24.45	30.17	15.79	9.32	14.36

Fig.4D

	WT+normoxia			Tg+normoxia			WT+hypoxia			Tg+hypoxia		
6 hr	0.16959	0.12366	0.14744	0.05	0.09043	0.02469	0.18878	0.13757	0.15847	0.11458	0.10204	0.08287
12 hr	0.4152	0.39891	0.25	0.23125	0.34574	0.08642	0.44898	0.45503	0.31148	0.24479	0.30102	0.1989

Fig.4E

mRNA	WT+saline			Tg+saline			WT+MCT			Tg+MCT		
TGF-β	0.94462	1.07758	0.97793	0.57347	0.46905	0.45939	1.77498	1.62203	1.39262	1.26383	0.59783	0.6915
TGFBR2	1.15337	0.70021	1.14541	0.44315	0.51258	0.60118	4.87655	3.08626	2.91978	0.64432	2.78149	0.59702
β-catenin	1.07986	0.92713	0.99367	0.70263	0.72741	0.71244	4.52724	7.29866	6.82506	1.09493	1.66577	0.45089
CTGF	1.09785	0.93192	0.96479	0.88778	0.53155	0.42877	21.5345	24.0911	26.3574	4.03066	2.7362	2.31062
IL-1β	0.86612	1.04438	1.08946	1.03717	1.07374	0.2611	4.06329	8.64968	4.06329	1.57018	0.96771	0.75401
ET-1	1.18139	1.00348	0.81508	0.11623	0.13822	0.35479	2.07774	2.36934	2.04793	1.22158	0.35038	1.5239
protein	WT+saline			Tg+saline			WT+MCT			Tg+MCT		
TGF-β	1	1	1	0.84918	0.87389	0.84611	2.14741	2.17374	1.89702	1.26617	1.25488	1.2219
TGFBR2	1	1	1	0.91994	0.9125	0.91623	2.54314	2.64316	2.65348	1.46063	1.38641	1.63575
β-catenin	1	1	1	0.68886	0.76615	0.88735	2.73166	3.39571	3.78264	1.01764	1.64165	1.78174
CTGF	1	1	1	0.69427	0.82768	0.7223	2.69145	3.87262	3.7899	0.96096	1.67758	1.71575
IL-1β	1	1	1	0.87986	0.88997	0.89927	2.76203	2.98531	3.35474	1.0372	1.69303	1.44149
ET-1	1	1	1	0.72527	0.72527	0.86406	1.87982	2.14959	1.71951	1.31067	1.29086	1.30373

Fig.4F

	WT+saline			Tg+saline			WT+MCT			Tg+MCT		
sera miR-483-3p	0.96635	1.0872	0.94646	2.49772	4.59675	3.75969	0.2969	0.29078	0.59524	1.96787	1.96301	1.60291
sera miR-483-5p	0.91498	1.29398	0.79104	2.46539	5.94558	1.79231	0.54271	0.6782	0.32126	1.58167	1.73659	1.57903
lung miR-483-3p	0.96561	1.04212	0.99276	2.55824	2.39416	2.69357	0.58084	0.61724	0.72074	2.94453	2.37204	2.49583
lung miR-483-5p	0.88622	0.88622	1.22751	5.01322	3.46086	4.2694	0.29642	0.42506	0.31116	4.27752	2.91168	4.27444

Fig.4G

	WT+saline			Tg+saline			WT+MCT			Tg+MCT		
Ago1-miR-483-3p	1.05098	1.2412	0.70796	3.53507	2.43132	2.89134	0.45747	0.37158	0.33028	3.09887	2.28428	2.73538
Ago1-miR-483-5p	0.9655	1.4234	0.61104	3.48061	5.89439	6.1447	0.22058	0.05749	0.45042	4.37518	4.11058	6.96123
Ago2-miR-483-3p	0.86595	1.26783	0.86595	3.84334	3.73825	4.47647	0.47053	0.36157	0.37432	3.1002	5.54952	5.39776
Ago2-miR-483-5p	0.88309	1.1572	0.95969	5.62027	5.94073	3.38848	0.3058	0.37648	0.62879	3.73379	6.06555	5.13598

Fig.4H

	WT+saline			Tg+saline			WT+MCT			Tg+MCT		
Ago1-TGF-β	1.27396	0.99045	0.73545	6.94982	0.8737	2.19919	0.40802	0.11318	0.18882	1.43095	5.62724	2.59675
Ago1-TGFBR2	0.94135	0.64752	1.41114	3.65408	2.97119	1.00633	0.27442	0.26325	0.29141	3.68524	2.22754	0.80409
Ago1-β-catenin	1.02453	0.8919	1.08357	2.5579	3.6116	2.25786	0.49481	0.44773	0.46764	3.3287	5.02422	2.15269
Ago1-CTGF	1.78863	0.54294	0.66843	5.85171	5.9334	4.86586	0.06881	0.01818	0.13275	4.67985	5.27242	4.38542
Ago1-IL-1β	1.1137	1.01284	0.87355	1.80694	2.29973	1.12976	0.36634	0.33772	0.62192	2.01576	2.69749	1.88635
Ago1-ET-1	0.96254	1.02797	1.00949	1.64817	2.56392	2.52565	0.64624	0.74733	0.59549	2.10134	1.26785	2.36969
Ago2-TGF-β	1.70792	0.89829	0.39378	13.6634	19.7289	16.168	0.1794	0.14183	0.29126	10.9453	12.9263	11.0324
Ago2-TGFBR2	0.55166	1.22476	1.22359	2.31028	1.3878	1.54878	0.1865	0.33828	0.26463	1.89025	1.9578	2.42432
Ago2-β-catenin	1.1537	0.89155	0.95475	2.81744	5.28077	3.44472	0.26506	0.33948	0.16576	3.56819	6.12362	3.39614
Ago2-CTGF	1.01186	1.08196	0.90618	2.13268	2.48588	1.89562	0.59985	0.50093	0.5875	2.0264	1.99404	1.75306
Ago2-IL-1β	0.81405	1.03157	1.15439	5.49056	0.53034	0.9689	0.43471	0.5152	0.11141	1.9052	2.50874	3.04222
Ago2-ET-1	0.99567	1.02206	0.98228	2.39376	2.33658	1.84435	0.05445	0.04087	0.02338	1.26458	2.17129	1.02104

All data were fold changes, normalized to "WT" (A, B), "WT+normoxia" (C, D), "WT+saline" (E-H).
