# Supplementary material for: MicroRNA‐483 amelioration of experimental pulmonary hypertension
Source: EMBO Mol Med. 2020 Apr 23;12(5):e11303. doi: 10.15252/emmm.201911303 (PMC7207157; doi:10.15252/emmm.201911303)
Supplement: Supplementary file 8 — Source Data for Figure 5 [file EMMM-12-e11303-s006.pdf]

WT+saline

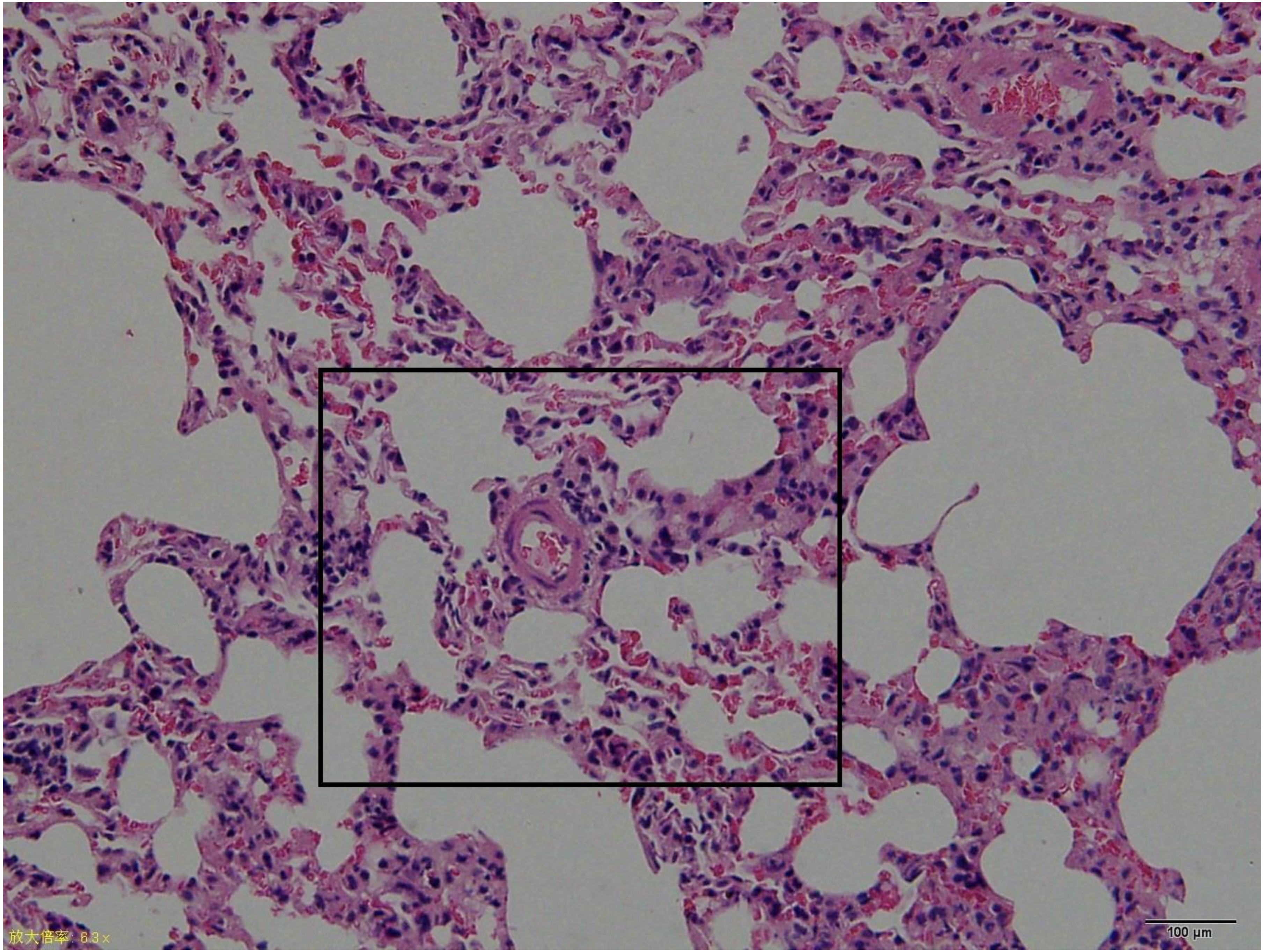

Tg+saline

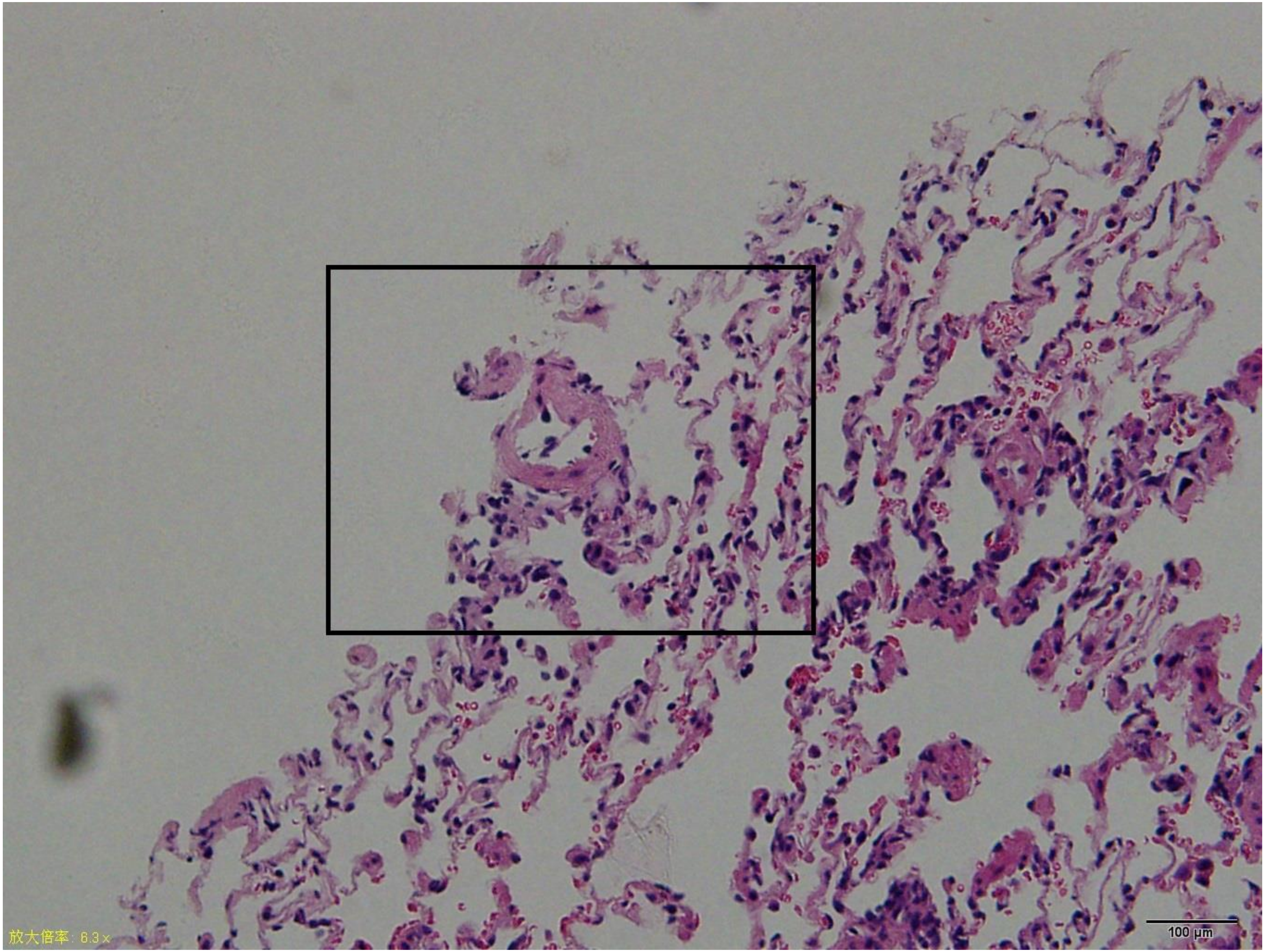

WT+MCT

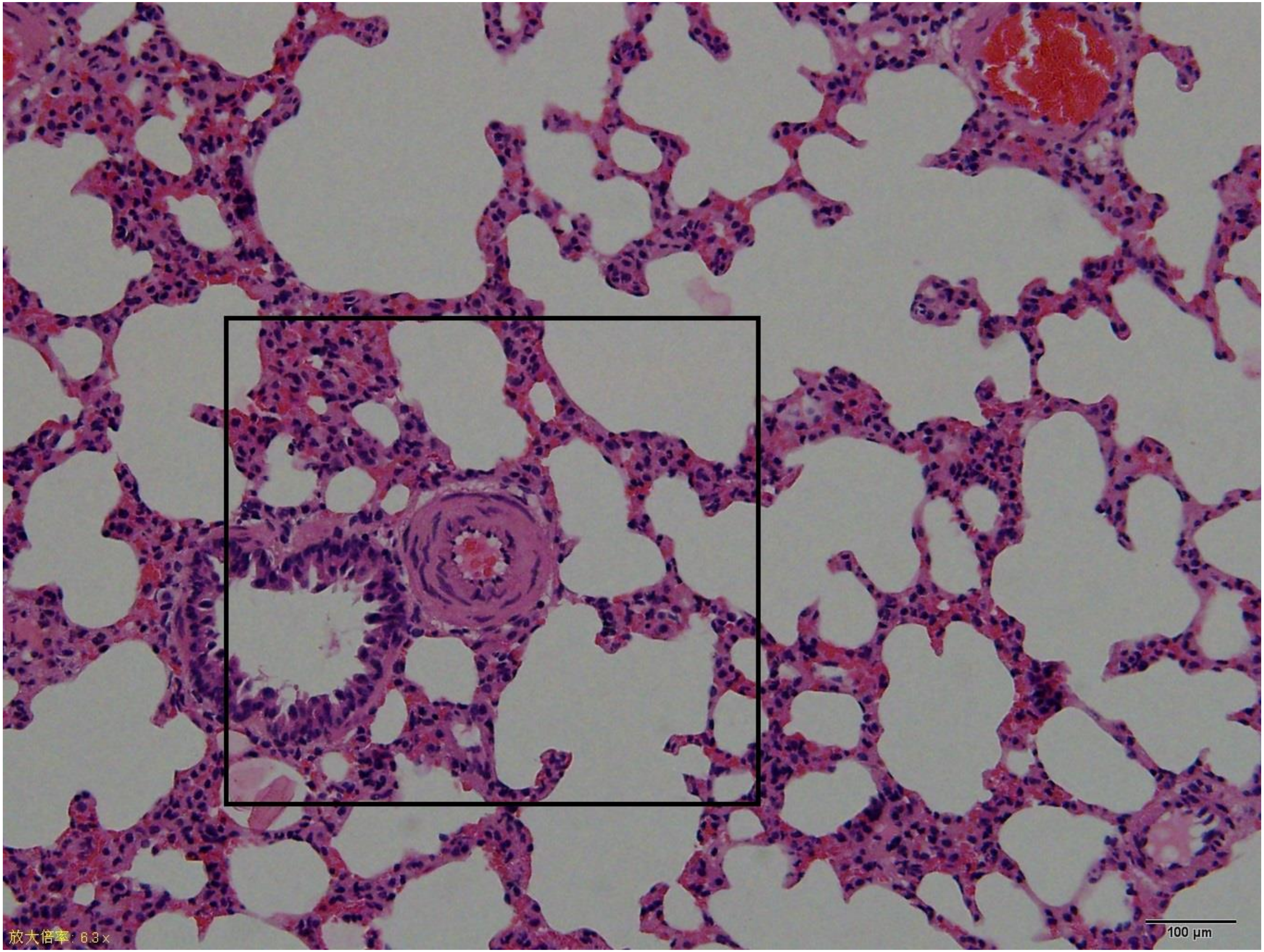

Tg+MCT

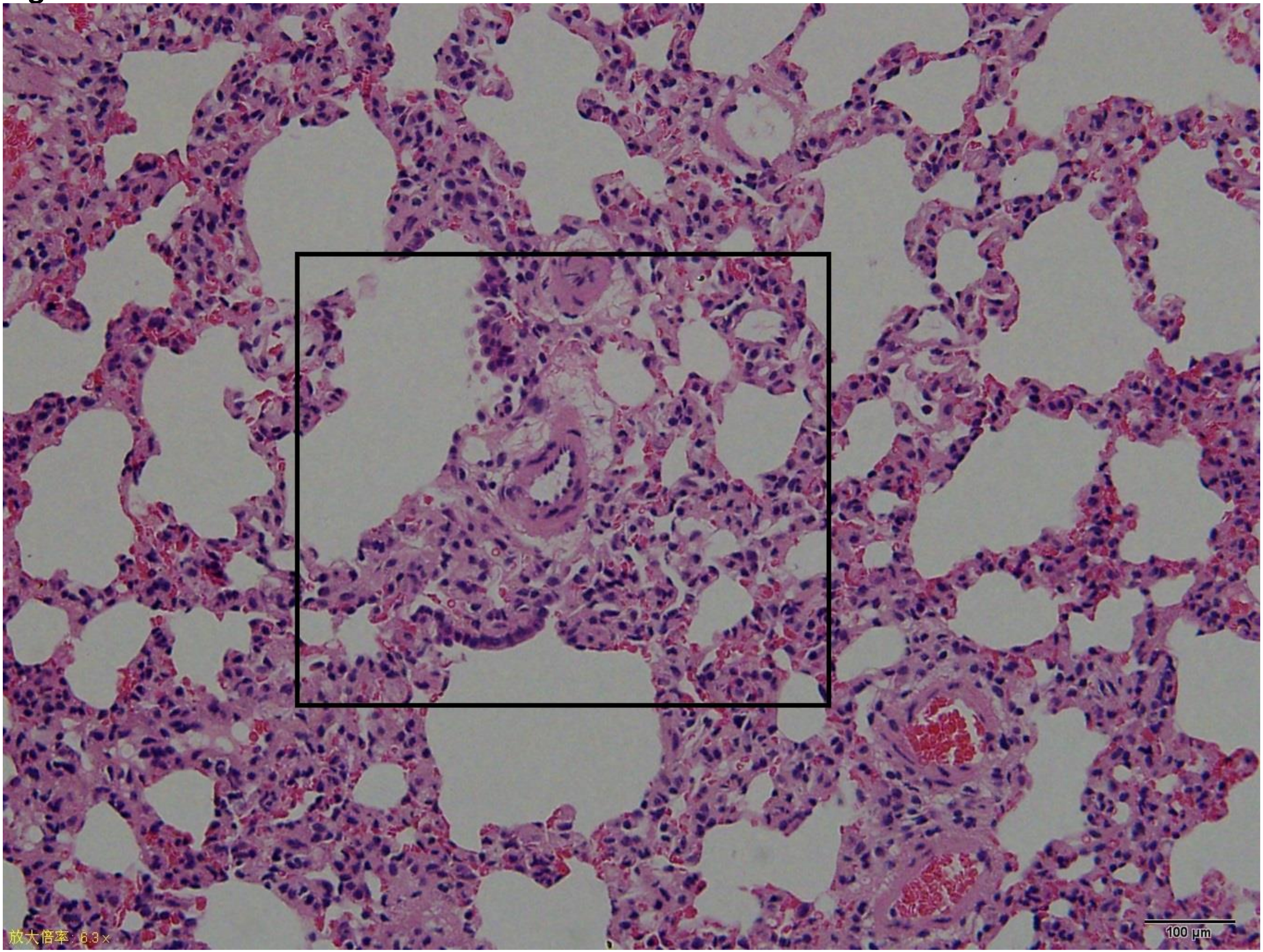

Fig.5A

WT+saline	Tg+saline	WT+MCT	Tg+MCT
20.832	27.775	42.29	21.2335
20.874	27.61533	34.87333	21.01167
29.38	26.70667	38.53933	31.1595
22.58467	22.76447	41	30.16933
16.22733	24.71867	38	29.70111
21.14667	22.1896	44	32.75555
24.35	22.0184	52.85333	31.9

Fig.5B

WT+saline	Tg+saline	WT+MCT	Tg+MCT
0.27381	0.227273	0.521277	0.322034
0.19802	0.253333	0.671642	0.390625
0.242424	0.242424	0.587302	0.4375
0.263889	0.313433	0.6375	0.244186
0.253521	0.267606	0.539474	0.348315
0.261539	0.292683	0.587302	0.333333
0.242857	0.229508	0.62069	0.297297

Fig.5C

WT+saline	Tg+saline	WT+MCT	Tg+MCT
29.6	25.2	69.5	42.7
24.9	24.4	73.9	36.2
25.2	27.6	81.7	47.6
23.1	25.7	76.7	33.9
28.1	26.8	71.5	38.7
25.9	23.4	70.5	44.4
24.7	21.9	74.7	41.3

Fig.5D

WT+saline	Tg+saline	WT+MCT	Tg+MCT
13.59019	28.2845	139.8477	35.34501
12.99384	18.07727	118.3607	61.45921
12.3851	22.84689	169.1029	48.59597
12.41929	23.17809	113.6511	21.6892
27.74891	27.64032	140.1476	9.5611
45.30716	9.689973	86.46706	40.29852
13.33588	22.79566	77.5337	49.40901

Fig.5E

WT+normoxia	Tg+normoxia	WT+SU/Hypoxia	Tg+SU/Hypoxia
20.73778	20.7	40.77667	24.75033
21.98033	18.2	39.12333	28.60775
22.49	25.37	41.65	26.38778
18.41	17.14	42.65	22.3
22.48	18.73	31.77	28.38
20	19.01	39.335	24.23
17.43	21.41	38.185	24.88

Fig.5F

WT+normoxia	Tg+normoxia	WT+SU/Hypoxia	Tg+SU/Hypoxia
0.254546	0.244444	0.440678	0.318182
0.277778	0.245283	0.42623	0.380952
0.241379	0.33871	0.344828	0.321429
0.226191	0.254237	0.363636	0.34
0.258065	0.166667	0.350877	0.169231
0.275862	0.259259	0.450704	0.361111
0.216667	0.261539	0.380952	0.402597

All data were fold changes, normalized to "WT+saline" (A-D), "WT+normoxia" (E, F).
