# Supplementary material for: MicroRNA‐483 amelioration of experimental pulmonary hypertension
Source: EMBO Mol Med. 2020 Apr 23;12(5):e11303. doi: 10.15252/emmm.201911303 (PMC7207157; doi:10.15252/emmm.201911303)
Supplement: Supplementary file 9 — Source Data for Figure 6 [file EMMM-12-e11303-s007.pdf]

Fig.6D

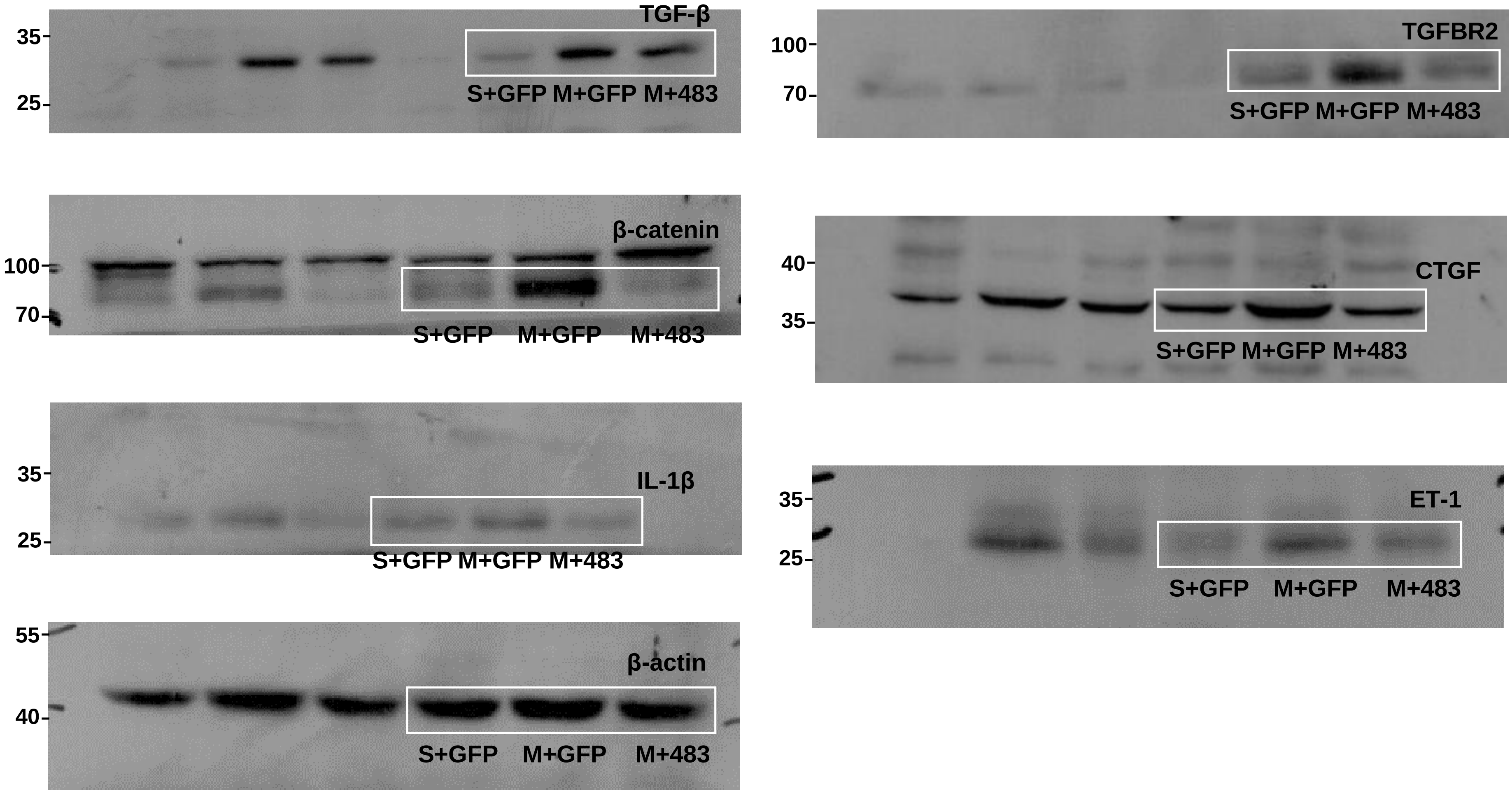
TGF-β
35
25
S+GFP M+GFP M+483
TGFBR2
100
70
S+GFP M+GFP M+483
β-catenin
100
70
S+GFP M+GFP M+483
CTGF
40
35
S+GFP M+GFP M+483
IL-1β
35
25
S+GFP M+GFP M+483
ET-1
35
25
S+GFP M+GFP M+483
β-actin
55
40
S+GFP M+GFP M+483

Saline+GFP

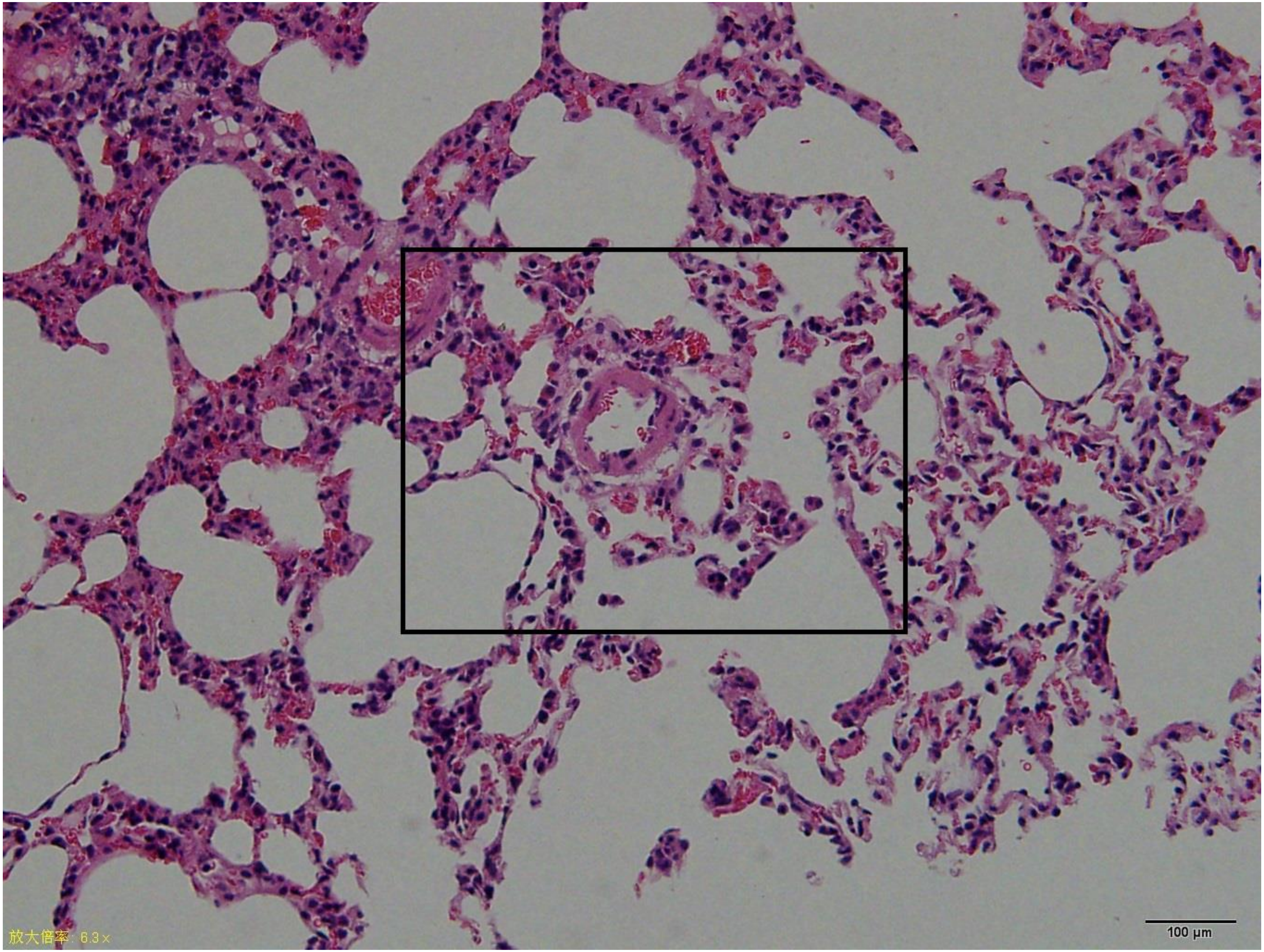

MCT+GFP

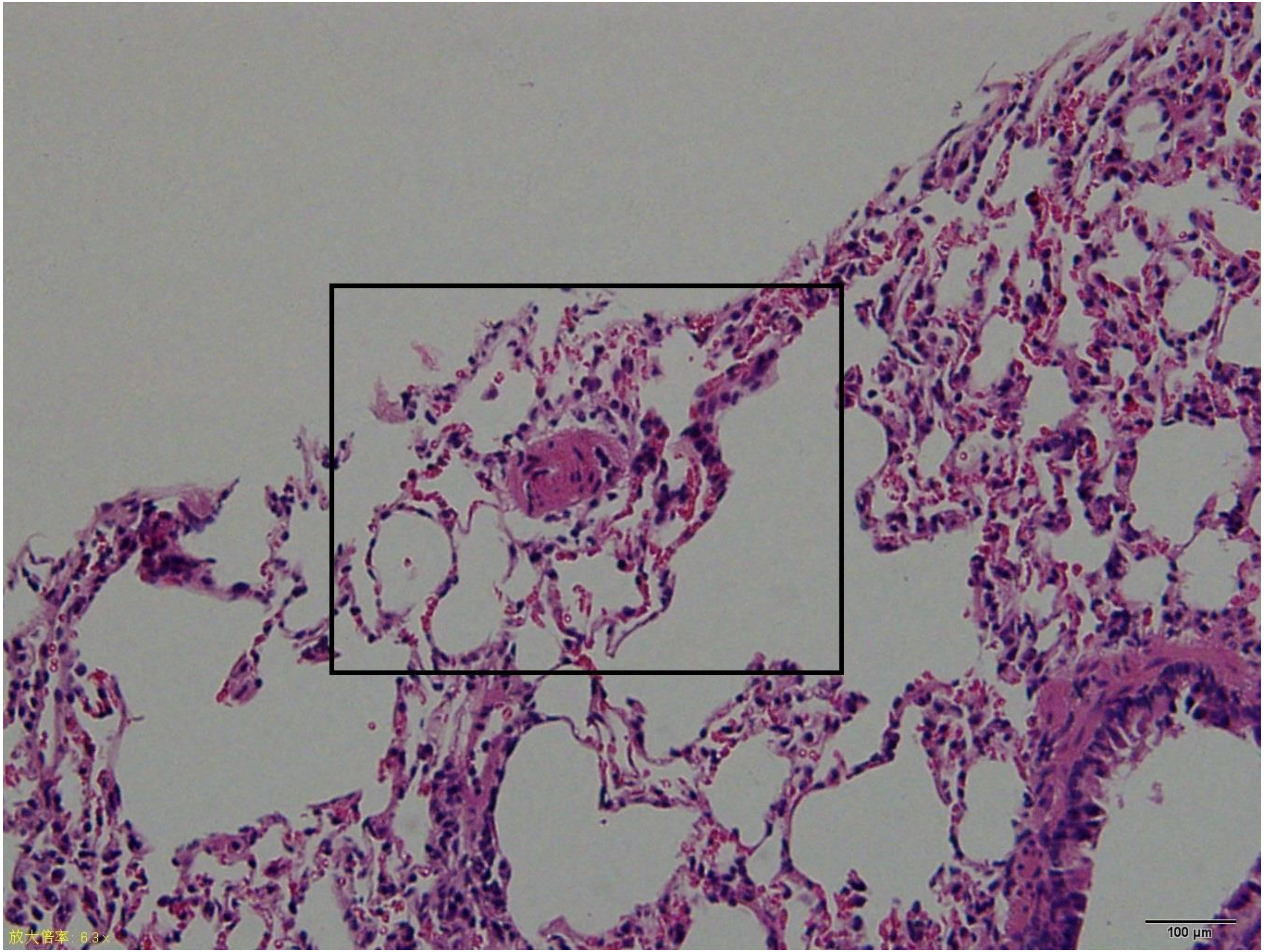

MCT+483

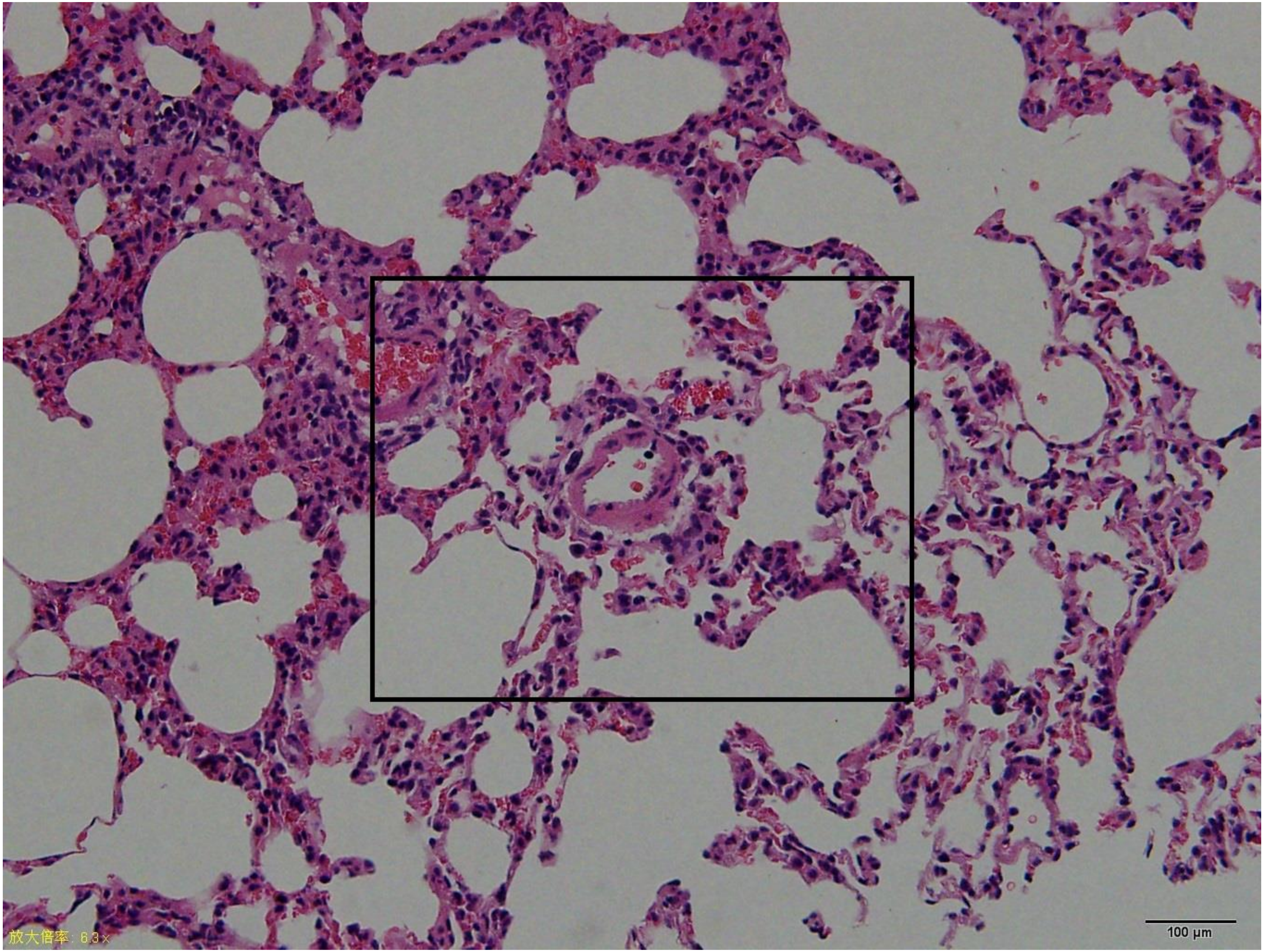

Fig.6B

	S+GFP			M+GFP			M+483		
sera-miR-483-3p	1.139	0.923	0.94	0.923	0.777	0.616	2.816914	3.446602	2.578982
sera-miR-483-5p	0.905389	0.888025	1.206433	0.554214	0.58851	0.640393	1.452431	1.526647	1.452124

Fig.6C

	S+GFP			M+GFP			M+483		
lung-miR-483-3p	1.148694	0.935699	0.915513	0.882482	0.735511	0.742083	2.347867	2.116016	3.163126
lung-miR-483-5p	0.96707	1.10411	0.928685	0.736681	0.798639	0.712926	2.77461	2.553162	4.062266

Fig.6D

mRNA	S+GFP			M+GFP			M+483		
TGF-β	0.894121	1.093188	1.012935	5.364607	5.073564	3.092002	1.44655	1.100792	1.163556
TGFBR2	0.791674	1.016055	1.191665	6.106955	5.035599	6.466469	0.385013	0.599977	0.349395
β-catenin	0.951639	1.041372	1.005899	13.16842	8.273432	7.404929	0.772971	0.998951	1.327292
CTGF	1.478401	0.891334	0.630268	21.31855	24.65892	23.32876	1.209187	1.217598	1.437974
IL-1β	1.462462	0.811352	0.72618	2.546294	2.022002	3.36973	0.706323	0.440862	0.577703
ET-1	0.722891	1.129968	1.14712	4.308467	3.595718	3.301362	0.611988	0.732672	1.289444
protein	S+GFP			M+GFP			M+483		
TGF-β	1	1	1	1.624241	1.752042	1.625364	0.989612	1.140238	1.243848
TGFBR2	1	1	1	1.492212	1.955886	1.802309	0.445515	0.375161	0.715866
β-catenin	1	1	1	2.298001	2.304834	1.762392	1.033481	0.957932	0.79128
CTGF	1	1	1	1.588432	1.854483	1.414226	1.027321	1.084248	0.90278
IL-1β	1	1	1	1.380245	1.512943	1.407722	0.74123	1.208815	0.695315
ET-1	1	1	1	2.36198	2.582919	1.673601	1.578444	1.897702	1.044675

Fig.6E

S+GFP	M+GFP	M+483
20.29	41	33.08
24.1	61.76	28.284
23.25	52.41	28.35
20.74	44.685	34.26
25.79	50.16067	28.94
25.35	40.548	23.33133
20.026	41.41611	26.87806

Fig.6F

S+GFP	M+GFP	M+483
0.233333	0.571429	0.520548
0.213333	0.544872	0.289855
0.25	0.627451	0.405063
0.243243	0.484849	0.458824
0.25	0.734483	0.385542
0.261628	0.524324	0.454546
0.260582	0.787671	0.525802

Fig.6G

S+GFP	M+GFP	M+483
95	126	120
99	130	115
120	118	117
96	110	106
99	101	92
120	93	93
133	95	110

Fig.6H

S+GFP	M+GFP	M+483
43.4783	71.5686	39.2157
28.5156	102.7397	58.1395
40	80	56.5217
39.4737	81.8182	51.9149
41.6667	87.0647	59.3103
40.2985	95	49.3724
42.9293	78.8177	55

Fig.6I

S+GFP	M+GFP	M+483
24.8	66.6	42.2
25.3	71.5	50.8
21.6	65.9	43
24.2	63.6	41
36.2	71.4	46.4
33.7	69.1	44.8
30.9	74.1	50.7

All data were fold changes, normalized to "S+GFP".
